# The Regulation of Pea (*Pisum sativum* L.) Symbiotic Nodule Infection and Defense Responses by Glutathione, Homoglutathione, and Their Ratio

**DOI:** 10.3389/fpls.2022.843565

**Published:** 2022-03-30

**Authors:** Kira A. Ivanova, Ekaterina N. Chernova, Olga A. Kulaeva, Anna V. Tsyganova, Pyotr G. Kusakin, Iana V. Russkikh, Igor A. Tikhonovich, Viktor E. Tsyganov

**Affiliations:** ^1^Laboratory of Molecular and Cellular Biology, Department of Biotechnology, All-Russia Research Institute for Agricultural Microbiology, Saint Petersburg, Russia; ^2^Saint Petersburg Federal Research Center of the Russian Academy of Sciences, Scientific Research Centre for Ecological Safety of the Russian Academy of Sciences, Saint Petersburg, Russia; ^3^Laboratory of Genetics of Plant-Microbe Interactions, Department of Biotechnology, All-Russia Research Institute for Agricultural Microbiology, Saint Petersburg, Russia; ^4^Department of Genetics and Biotechnology, Saint Petersburg State University, Saint Petersburg, Russia; ^5^Saint Petersburg Scientific Center of the Russian Academy of Sciences, Saint Petersburg, Russia

**Keywords:** *Rhizobium*–legume symbiosis, bacteroid, infection droplet, infection thread, symbiosome, γ-glutamylcysteine synthetase, glutathione synthetase, homoglutathione synthetase

## Abstract

In this study, the roles of glutathione (GSH), homoglutathione (hGSH), and their ratio in symbiotic nodule development and functioning, as well as in defense responses accompanying ineffective nodulation in pea (*Pisum sativum*) were investigated. The expression of genes involved in (h)GSH biosynthesis, thiol content, and localization of the reduced form of GSH were analyzed in nodules of wild-type pea plants and mutants *sym33-3* (weak allele, “locked” infection threads, occasional bacterial release, and defense reactions) and *sym33-2* (strong allele, “locked” infection threads, defense reactions), and *sym40-1* (abnormal bacteroids, oxidative stress, early senescence, and defense reactions). The effects of (h)GSH depletion and GSH treatment on nodule number and development were also examined. The GSH:hGSH ratio was found to be higher in nodules than in uninoculated roots in all genotypes analyzed, with the highest value being detected in wild-type nodules. Moreover, it was demonstrated, that a *hGSHS*-to-*GSHS* switch in gene expression in nodule tissue occurs only after bacterial release and leads to an increase in the GSH:hGSH ratio. Ineffective nodules showed variable GSH:hGSH ratios that correlated with the stage of nodule development. Changes in the levels of both thiols led to the activation of defense responses in nodules. The application of a (h)GSH biosynthesis inhibitor disrupted the nitrogen fixation zone in wild-type nodules, affected symbiosome formation in *sym40-1* mutant nodules, and meristem functioning and infection thread growth in *sym33-3* mutant nodules. An increase in the levels of both thiols following GSH treatment promoted both infection and extension of defense responses in *sym33-3* nodules, whereas a similar increase in *sym40-1* nodules led to the formation of infected cells resembling wild-type nitrogen-fixing cells and the disappearance of an early senescence zone in the base of the nodule. Meanwhile, an increase in hGSH levels in *sym40-1* nodules resulting from GSH treatment manifested as a restriction of infection similar to that seen in untreated *sym33-3* nodules. These findings indicated that a certain level of thiols is required for proper symbiotic nitrogen fixation and that changes in thiol content or the GSH:hGSH ratio are associated with different abnormalities and defense responses.

## Introduction

The plastic responses of legumes to nitrogen-limited conditions through the formation of nitrogen-fixing nodules merits extensive investigation. The development of indeterminate symbiotic nodules (with prolonged meristem activity) involves processes such as root hair deformations, infection thread growth, the reactivation of cell division in the root inner cortex and pericycle followed by nodule primordium development, bacterial release, the differentiation of infected cells associated with repeated rounds of endoreduplication, and the differentiation of bacteria into nitrogen-fixing bacteroids (Tsyganova et al., 2018). These processes are accompanied by the activation and suppression of plant defense responses (Gourion et al., 2015; Benezech et al., 2020). Mature indeterminate nodules are characterized by a distinct zonation, namely, a meristematic zone, an infection zone, a nitrogen fixation zone, and, finally, a senescence zone (Hirsch, 1992; Guinel, 2009). Nodule development is also linked to reactive oxygen species production (Puppo et al., 2005, 2013; Pauly et al., 2006), indicating that the antioxidant system has a role in the formation and functioning of nitrogen-fixing nodules (Ivanova and Tsyganov, 2017). Interactions between pro- and antioxidant factors regulate the redox state of a plant cell and that of its compartments, thereby controlling a variety of cellular processes (Ribeiro et al., 2015). Low-molecular-weight thiol glutathione (GSH) is a water-soluble antioxidant present in plant nodules and functions as a redox buffer. GSH and its legume-specific homolog homoglutathione (hGSH) are involved in plant developmental processes and stress adaptation, including nodulation (Noctor et al., 2012; Bianucci et al., 2017; Boncompagni et al., 2017; Locato et al., 2017; Matamoros and Becana, 2020).

The first rate-limiting step in the synthesis of both GSH and hGSH is the formation of γ-glutamylcysteine (γ-Glu-Cys) catalyzed by γ-glutamylcysteine synthetase (γ-ECS, encoded by *GSH1*). The second step in GSH synthesis is catalyzed by glutathione synthetase (encoded by *GSHS*) and involves the addition of glycine to γ-Glu-Cys (Frendo et al., 2001); in contrast, instead of glycine, hGSH synthesis involves the addition of β-alanine to γ-Glu-Cys, a process catalyzed by the enzyme homoglutathione synthetase encoded by the *hGSHS* gene (Frendo et al., 2001; Iturbe-Ormaetxe et al., 2002). Despite there being only one amino acid difference between GSH and hGSH, specific *cis*-regulatory elements are present in the promoter regions of both *GSHS* and *hGSHS*, indicating that, in legumes, they may either perform different functions or perform similar functions that are separated temporally or spatially (Clemente et al., 2012). Indeed, hGSH tends to accumulate in legume leaves and roots, whereas GSH is usually present at high concentrations in seeds (Colville et al., 2015). In *Medicago sativa*, GSH is associated with meristematic cells, cell-cycle activation, and the induction of somatic embryogenesis, while hGSH is associated with differentiated cells and embryo proliferation (Pasternak et al., 2014); in *Lotus japonicus*, meanwhile, *GSHS* is expressed only in nodules, whereas *hGSHS* is also expressed in leaves and roots (Matamoros et al., 2003); and in *M*. *truncatula*, *hGSHS* is expressed in roots and nodules while *GSHS* is expressed in all tissues (Frendo et al., 1999). *GSHS* and *hGSHS* expression in legumes is also differentially regulated in response to signaling molecules or stress (Innocenti et al., 2007; Loscos et al., 2008; Becana et al., 2010). Increased GSH levels can lead to increased nitrogen fixation efficiency; however, no similar data exists for hGSH. Analysis of (h)GSH distribution in tissues of 73 species from the Fabaceae family indicated that hGSH in roots is unrelated to nodulation (Colville et al., 2015). Little difference was found in the patterns of hGSH responses to hormone treatments between nodulated and non-nodulated plants (Clemente et al., 2012). Nevertheless, the exact function of hGSH in nodules remains unknown. Here, we undertook a comparative analysis of effective and ineffective pea symbiotic nodules to test the involvement of both thiols in nodule development and functioning, as well as in plant defense responses triggered by plant symbiosis-related mutations.

## Materials and Methods

### Plant Material, Bacterial Strain, and Plant Growth Conditions

The pea (*Pisum sativum* L.) laboratory line SGE and corresponding mutant lines blocked at different stages of nodule development were analyzed (Table 1). Plant growth conditions were as previously described (Kitaeva et al., 2016). Nodules were harvested and frozen in liquid nitrogen 1, 2, or 3 weeks post-inoculation for gene expression and high-performance liquid chromatography–mass spectrometric (HPLC–MS) analyses. The roots of 1, 2, and 3-week-old uninoculated plants were used as controls. The material was stored at −80°C.

**TABLE 1:** Plant material used in the study.

Genotype	Nodule phenotype	References
SGE	Wild type	Kosterin and Rozov (1993)
(*sym40-1*)*	Hypertrophied infection droplets, abnormal bacteroids, oxidative stress inside the nodule, activation of defense response genes, early nodule senescence phenotype	Tsyganov et al. (1998); Tsyganova et al. (2009); Ivanova et al. (2015)
(*sym33-3*)**, weak allele	“Locked” suberinized infection threads, increase in deposition of unesterified pectin in infection thread walls, absence of bacterial release into the host cell cytoplasm of most infected cells***, activation of defense response genes, deposition of cell wall material into vacuole.	Tsyganov et al. (1998); Voroshilova et al. (2001); Ivanova et al. (2015); Tsyganova et al. (2019b)
(*sym33-2*)**, strong allele	“Locked” suberinized infection threads and the absence of bacterial release; in some of these infection threads, bacteria form clusters and show signs of degradation.	Tsyganov et al. (1998); Voroshilova et al. (2001); Tsyganova et al. (2019a)

*The Sym40 gene is orthologous to the M. truncatula EFD gene (Tsyganov and Tsyganova, 2020).

**The Sym33 gene is orthologous to the M. truncatula IPD3 gene (Tsyganov and Tsyganova, 2020).

***The mutant line sym33-3 has a leaky phenotype and bacterial release occurs in some cells or nodules (Tsyganov et al., 1998; Voroshilova et al., 2001).

### Fixation, Immunolabeling, and Microscopy

Three-week-old nodules were fixed and prepared for immunolabeling as previously described (Kitaeva et al., 2016). For immunolocalization, a rabbit anti-GSH (reduced) antibody (Agrisera, Vännäs, Sweden) was diluted 1:100 in TBS (50 mM Tris-HCl, 150 mM NaCl, pH 7.5) containing 2 mg mL^–1^ BSA and 0.4 mg mL^–1^ BSA-C. Goat anti-rabbit IgG Alexa Fluor 488 (Thermo Fisher Scientific, Waltham, MA, United States) diluted 1:500 in TBS was used as a secondary antibody. Sections were also stained with propidium iodide (0.5 μg mL^–1^) to visualize nuclei and bacteria. Sections were mounted in ProLong Gold antifade reagent (Thermo Fisher Scientific) and analyzed using confocal laser scanning microscopy (LSM 510 META and LSM 780, Zeiss, Oberkochen, Germany).

### Immunogold Labeling, Transmission Electron Microscopy, and Quantitative Analysis

Samples were prepared as previously described (Serova et al., 2019). Sections were treated with the primary rabbit anti-GSH (reduced) antibody (Agrisera) diluted 1:25 in 0.1% BSA-C in PBS (136 mM NaCl, 2.68 mM KCl, 10 mM Na_2_HPO_4_, 1.7 mM KH_2_PO_4_, pH 7.4) at 4°C overnight. After four rinses in 0.1% BSA-C in PBS, the samples were incubated with a 10-nm gold-conjugated secondary goat anti-rabbit IgG antibody (Amersham International, Little Chalfont, United Kingdom) diluted 1:50 in 0.1% BSA-C in PBS for 4 h at 37°C. Labeled grids were post-contrasted with uranyl acetate for 15 s. Negative controls were treated either with pre-immune serum instead of the primary antibody (Supplementary Figure 1A) or non-specific secondary antibody (goat anti-mouse IgG) (Supplementary Figure 1B). Nodule tissues were analyzed using a JEM–1400 EM transmission electron microscope (80 kV) (JEOL Ltd, Tokyo, Japan) equipped with a Veleta CCD camera (Olympus, Münster, Germany). Micrographs of randomly imaged immunogold-labeled sections were digitized and gold particles were quantified in visually identified cell structures. For statistical analysis, at least 15 nodule samples and 50 sectioned symbiosomes were examined for the wild-type and mutant lines (for *sym33-3*, mutant nodules with bacterial release were analyzed). Morphometrical data were obtained as previously described (Ivanova et al., 2015). The number of gold particles per unit area was measured using Zen 2 Core version 2.5 software (Zeiss). Twenty nodules from at least five plants were analyzed for each genotype.

### Total RNA Isolation, cDNA Synthesis, and Relative Real-Time PCR

RNA isolation, cDNA synthesis, and relative real-time PCR were performed as previously described (Serova et al., 2019). Glyceraldehyde-3-phosphate dehydrogenase (*GAPC1*) was used as a constitutively expressed reference gene (Ivanova et al., 2015). One- or three-week-old wild-type uninoculated roots were used as calibrator samples for relative transcript abundance calculation. Experiments were performed with three replicates of a minimum of 10 plants per genotype. Primers (Supplementary Table 1) were designed using VectorNTI Advanced 10 software (Thermo Fisher Scientific).

### Selective Thiol Quantification Using High-Performance Liquid Chromatography–HRMS

Thiols were isolated as described by Bardarov et al. (2015) with some modifications. Samples were homogenized in liquid nitrogen using a mortar and pestle. Dithiotreitol (200 mM; Fluka Chemika, Buchs, Switzerland) was added to each 100 mg of plant tissue. Samples were disintegrated by sonication (Soniprep 150 Plus, MSE, Nuaillé, France) for 2 min on ice and incubated for 1.5 h at 4°C with vortexing every 20 min. The samples were then centrifuged (Centrifuge 5430R, Eppendorf, Germany) at 2500 × *g* for 8 min at 4°C. The supernatants were transferred into a new tube containing a SpinX HPLC 0.2-μm nylon microcentrifuge filter (Costar, Corning, NY, United States), centrifuged again at 10,000 × *g* for 2 min at 4°C, and stored at −80°C until used. Analysis was performed using the LC-20 Prominence HPLC system (Shimadzu, Kyoto, Japan) coupled to a LTQ Orbitrap XL Hybrid Ion Trap-Orbitrap Mass Spectrometer (Thermo Fisher Scientific) according to Chernova et al. (2018), with minor changes. Thiols were separated on a Supelcosil column (15 cm × 3.0 mm, 3 μm; Supelco, Bellefonte, PA, United States), with a guard column, by gradient elution (0.4 mL min^–1^) with a mixture of water and acetonitrile, both containing 0.1% formic acid. Mass spectrometric analysis was performed in positive electrospray ionization mode. Target compound identification was based on the accurate mass measurement of [M+N]^+^ ions (resolution: 30,000; accuracy within 5 ppm), the collected fragmentation spectra of the ions, and the retention times. Heater and capillary temperatures were both 300°C and the source voltage was 3.5 kV. The concentrations of the detected thiols were calculated based on the peak area of GSH and hGSH standards (SPC Verta, Saint Petersburg, Russia). Standard stock solutions (5 mM L^–1^) were prepared by dissolving in 250 mM dithiotreitol. GSH/hGSH amount in the samples were normalized relative to the GSH amount in 1- or 3-week-old wild-type nodules. Experiments were performed with four replicates, each with a minimum of 10 plants per genotype.

### L-Buthionine-Sulfoximine Treatment

L-Buthionine-sulfoximine (BSO) (Sigma-Aldrich, St. Louis, MO, United States) blocks (h)GSH synthesis by inhibiting γ-ECS (Griffith and Meister, 1979). To eliminate GSH, which is present in large amounts in seeds, cotyledons were removed from 3-day-old seedlings. Seedlings were divided into two sets and grown in sterile pots with 100 g of vermiculite and 200 mL of nitrogen-free medium (Fåhraeus, 1957) in the presence or absence (untreated plants) of 0.1 mM BSO in nutrient solution to decrease the thiol levels in treated plants. Ten-day-old seedlings were inoculated with rhizobia and transplanted into new pots with vermiculite supplemented with Fåhraeus nutrient solution. Plants previously treated with 0.1 mM BSO were subdivided into two groups. Plants from one subgroup were planted in pots and supplemented with a nutrient solution containing 0.1 mM BSO (BSO-treated plants). Those from the other subgroup were planted in pots supplemented with a nutrient solution containing 0.1 mM BSO and 0.5 mM GSH (BSO+GSH-treated plants); these plants should recover GSH, but not hGSH, in cells. All the plants were watered by weight with aqueous solutions containing the same concentrations of chemicals every 3 days. Plants were harvested 2 weeks post-inoculation and nodule numbers were counted. Twenty nodules per variant were collected for microscopy and the rest of the root system was frozen in liquid nitrogen for gene expression and HPLC–MS analysis.

### Glutathione Treatment

Pea seedlings were inoculated with rhizobia, divided into 3 sets, and grown in pots with 100 g of vermiculite and 200 mL of nitrogen-free medium. Plants were given water (untreated plants) or an aqueous solution containing GSH at a concentration of 0.1 or 1 mM starting from 64 h post-inoculation. All the plants were watered by weight with aqueous solutions containing the same concentrations of chemicals every 3 days. Plants were harvested 2 weeks post-inoculation. At least 20 nodules per variant were collected for microscopy while the rest of the root system was frozen in liquid nitrogen for gene expression and HPLC–MS analysis.

### Statistical Analysis

Immunogold labeling data were analyzed by *t*-tests corrected for multiple comparisons using R software (R Core Team^1^); GraphPad Prism (GraphPad Software, Inc., San Diego, CA, United States) software was used to compare gene expression (analysis of thiol biosynthesis genes: two-way ANOVA, analysis of gene expression in experiments with treatments: one-way ANOVA) and thiol levels (two-way ANOVA). In all comparisons a *P*-value < 0.05 was considered significant.

## Results

### Immunolocalization of the Reduced Form of Glutathione in Wild-Type and Mutant Nodules

**FIGURE 1:**
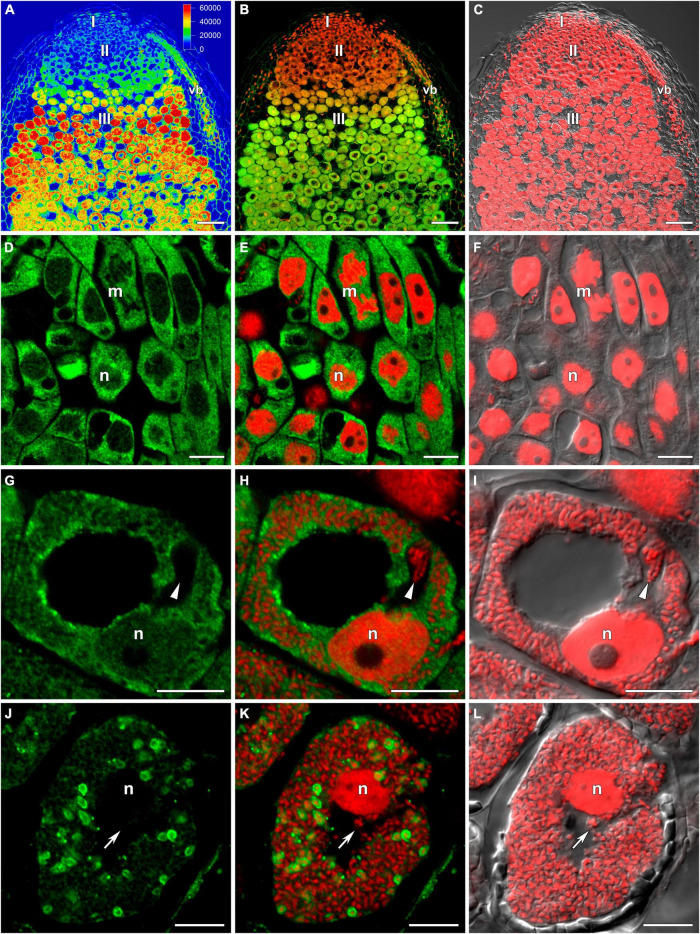
Immunolocalization of glutathione in 3-week-old nodules of wild-type pea (*Pisum sativum*) using a rabbit anti-glutathione (reduced) antibody. **(A–C)** General view; **(D–F)** meristem; **(G–I)** cells from the late infection zone; **(J–L)** infected cells from the nitrogen fixation zone. **(A)** Heatmap showing color-coded fluorescence signal intensities for the green channel; **(D,G,J)** green channel; **(B,E,H,K)** merge of the green and red channels; **(C,F,I,L)** merge of differential interference contrast and the red channel. Confocal laser scanning microscopy images of 50-μm-thick longitudinal sections. A single optical section is presented, glutathione in green and DNA (bacteria and nuclei) in red. I, meristematic zone; II, infection zone; III, nitrogen fixation zone; vb, vascular bundle; m, mitosis; n, nucleus. Arrows indicate infection threads; arrowheads indicate infection droplets. Scale bars = 100 μm **(A–C)** and 10 μm **(D–L)**.

In wild-type nodules (Figures 1A–C), GSH was detected in the cytoplasm and nuclei of meristematic cells (Figures 1D–F), the cytoplasm and nuclei of infected cells in the infection zone (Figures 1G–I), the cytoplasm and symbiosomes of cells in the nitrogen fixation zone (Figures 1J–L), and cells of the nodule parenchyma and vascular tissue (Figures 1A–C). The highest signal was observed in the symbiosomes of infected cells from the nitrogen fixation zone. The signal was absent or very weak in the matrix of infection threads and droplets (Figures 1G–L).

**FIGURE 2:**
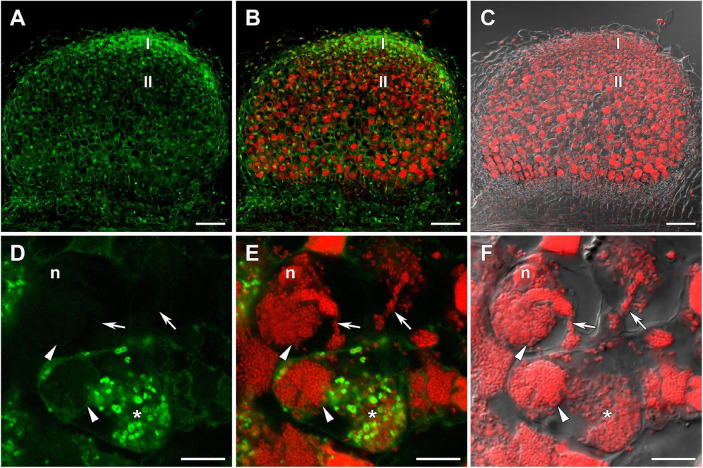
Immunolocalization of glutathione in 3-week-old nodules of the pea (*Pisum sativum*) *sym40-1* mutant using a rabbit anti-glutathione (reduced) antibody. **(A–C)** General view; **(D–F)** infected cells. **(A,D)** Green channel; **(B,E)** merge of the green and red channels; **(C,F)** merge of differential interference contrast and the red channel. Confocal laser scanning microscopy images of 50-μm-thick longitudinal sections. A single optical section is presented, glutathione in green and DNA (bacteria and nuclei) in red. I, meristematic zone; II, infection zone; n, nucleus; *, released bacteria. Arrows indicate infection threads; arrowheads indicate infection droplets. Scale bars = 100 μm **(A–C)** and 10 μm **(D–F)**.

**FIGURE 3:**
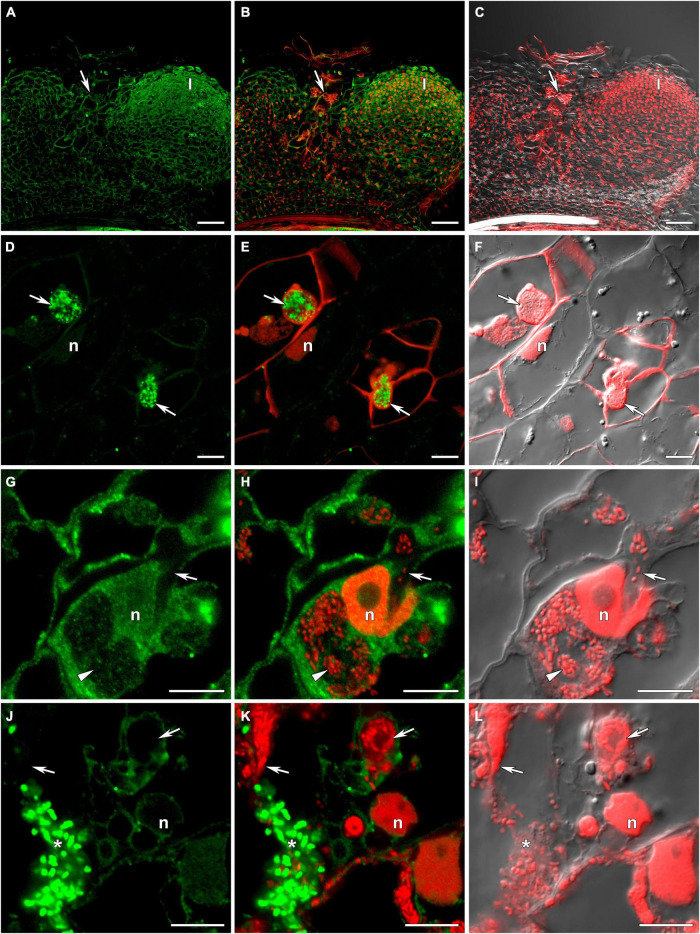
Immunolocalization of glutathione in 3-week-old nodules of the pea (*Pisum sativum*) *sym33-3* mutant using a rabbit anti-glutathione (reduced) antibody. **(A–C)** General view; **(D–F)** “locked” infection threads; **(G–I)** infected cells; **(J–L)** cells with released bacteria. **(A,D,G,J)** Green channel; **(B,E,H,K)** merge of the green and red channels; **(C,F,I,L)** merge of differential interference contrast and the red channel. Confocal laser scanning microscopy images of 50-μm-thick longitudinal sections. A single optical section is presented, glutathione in green and DNA (bacteria and nuclei) in red. I, meristematic zone; n, nucleus; *, released bacteria. Arrows indicate infection threads; arrowheads indicate infection droplets. Scale bars = 100 μm **(A–C)** and 10 μm **(D–L)**.

The distribution of GSH was also analyzed in symbiotic nodules of *sym40-1* (Figure 2), *sym33-2* (Supplementary Figure 2), and *sym33-3* (Figure 3) mutants. These mutants are defective in infection thread, infection droplet, and symbiosome development (Table 1). In *sym40-1* nodules, labeling was mainly associated with meristematic cells and the cytoplasm and nuclei of infected cells (Figures 2A–C), with the strongest signal being observed in bacteria released from infection droplets (Figures 2D–F). Similar to wild-type nodules, no signal was observed in the matrix of infection threads or hypertrophied infection droplets (Figures 2D–F).

The *sym33-2* mutant manifested a severe phenotype characterized by the formation of a highly branched network of “locked” infection threads without bacterial release that are arrested in the root outer cortex. Labeling of GSH was associated with the cytoplasm (Supplementary Figure 2).

The *sym33-3* mutant formed white nodules with “locked” infection threads without bacterial release (Figures 3A–C). In some of these infection threads, likely the first to penetrate the nodule primordium, the strongest signal was associated with bacteria (Figures 3D–F). In some cells of some nodules, infection threads formed infection droplets from which bacteria were released. No signal was observed in these infection threads but a signal could be detected in the cytoplasm of infected cells (Figures 3G–I). The strongest signal was associated with released bacteria and meristematic cells (Figures 3A–C,J–L).

### Immunogold Localization of the Reduced Form of Glutathione in the Infection Structures of Wild-Type and Mutant Nodules

**FIGURE 4:**
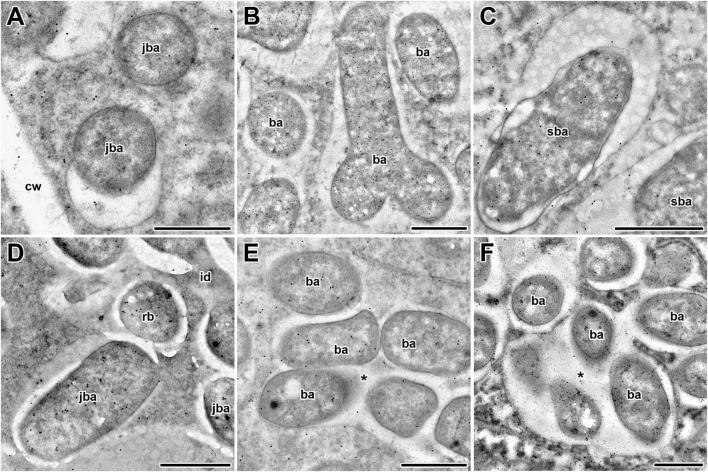
Immunogold localization of glutathione in symbiosomes of nodules from 2-week-old wild-type and mutant (*sym40-1* and *sym33-3*) pea (*Pisum sativum*) plants. **(A–C)** Wild type; **(D,E)** *sym40-1*; **(F)** *sym33-3*; **(A,D)** juvenile symbiosomes; **(B)** mature symbiosomes; **(C)** senescent symbiosomes; **(E,F)** abnormal symbiosomes. Transmission electron micrographs of 90-nm ultrathin sections labeled with goat anti-rat IgG monoclonal antibody conjugated to 10-nm-diameter colloidal gold. cw, cell wall; id, infection droplet; rb, releasing bacterium; ba, bacteroid; jba, juvenile bacteroid; sba, senescent bacteroid; * indicates several bacteroids surrounded by a common symbiosome membrane. Scale bar = 500 nm.

In wild-type nodules, gold particles were observed in nuclei, vacuoles, and plastids (data not shown). In infection threads and infection droplets, the signal was weak and mainly associated with bacteria (Supplementary Figures 3A,B). The amount of label was markedly greater in mature, nitrogen-fixing bacteroids than in juvenile bacteroids; however, labeling levels were similar between senescent and juvenile bacteroids (Figures 4A–C). Morphometric analysis showed that labeling for GSH was mainly associated with bacteroids and that the amount of label in the peribacteroid space remained constant throughout bacteroid differentiation (Table 2). The amount of GSH label in the cytoplasm of infected cells also remained constant, but was higher than that in the peribacteroid space (Table 2). In the *sym40-1* mutant, the amount of GSH label was higher in juvenile bacteroids than in wild-type ones (Figure 4D and Table 2); however, the amount of label was lower in the abnormal bacteroids of both the *sym40-1* and *sym33-3* mutants than in mature bacteroids of wild-type nodules (Figures 4E,F and Table 2). The amount of label was higher in the infection threads and droplets of the *sym40-1* mutant than in those of the *sym33-3* mutant in which bacterial release occurred (Supplementary Figures 3C,D and Table 2). Interestingly, in the cytoplasm of infected cells in ineffective mutants, especially the *sym33-3* mutant, the amount of GSH label was increased compared with that in the wild type (Table 2). This indicated that the amount of GSH increased significantly due to active nitrogen fixation in wild-type nodules and enhanced defense responses in the nodules of ineffective mutants (Ivanova et al., 2015; Tsyganova et al., 2019b).

**TABLE 2:** The distribution of immunogold-labeled glutathione in regions of infected cells in pea wild-type and mutant nodules.

Genotype	Type	Bacteroid	Peribacteroid space	Infection threads / droplets	Cytoplasm
	Juvenile	4.71 ± 0.41	6.3 ± 0.63^&^		8.94 ± 0.43
Wild type	Mature	78.59 ± 5.74^a^	3.42 ± 0.28^a,&^	3.93 ± 0.23	8.74 ± 0.73
	Senescent	7.52 ± 0.83^b^	4.6 ± 0.58^&^		7.9 ± 0.51

	Juvenile	24.71 ± 0.8^a,b,c^	8.07 ± 0.4^b,c,&^		
*sym40-1*				7.37 ± 0.31^a^	14.03 ± 0.91^a,b,c^
	Abnormal	6.25 ± 0.33^b,d^	7.89 ± 0.41^b,c,&^		

*sym33-3*	Abnormal	10.59 ± 0.48^b,d^	7.59 ± 0.33^b,c,&^	4.08 ± 0.22^d^	24.12 ± 1.01^a,b,c,d^

*Values are means ± SE. The results are presented as the number of gold particles in μm^–2^.*

*Letters indicate statistically significant differences (t-test corrected for multiple comparisons, padj < 0.05).*

*^a^From the juvenile wild-type (WT) cell region (or WT value of the same cell region).*

*^b^From the mature WT cell region.*

*^c^From the senescent WT cell region.*

*^d^From the juvenile sym40-1 cell region (or sym40-1 value of the same cell region).*

*^&^From the bacteroid value of the same genotype.*

### The Transcription Patterns of (Homo)Glutathione Biosynthesis-Related Genes and the Thiol Content in Wild-Type and Mutant Nodules and Uninoculated Roots

**FIGURE 5:**
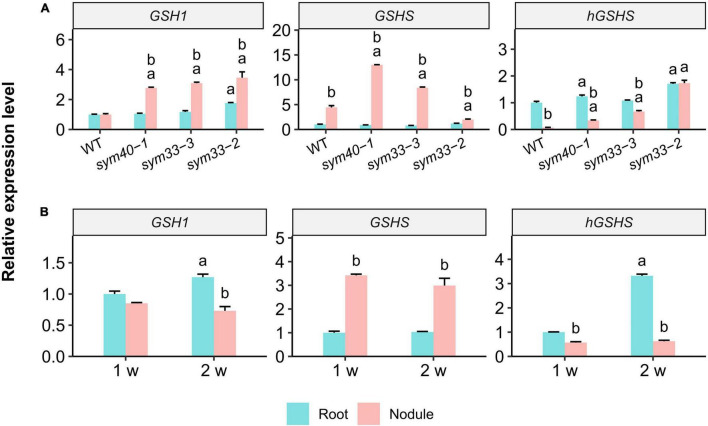
The relative expression levels of glutathione biosynthesis-related genes in nodules and roots of wild-type (WT) and mutant (*sym40-1*, *sym33-3*, and *sym33-2*) pea (*Pisum sativum*) plants. **(A)** Three-week-old uninoculated roots and nodules. **(B)** One- and two-week-old uninoculated roots and nodules of wild type. Transcript levels were determined by real-time PCR. Relative gene expression levels were quantified using the 2^–ΔΔCt^ method and normalized to those of the reference gene glyceraldehyde-3-phosphate dehydrogenase (*PsGAPC1*). Three-week-old **(A)** and 1-week-old **(B)** wild-type uninoculated (control) roots were used as calibrators for the calculation of relative transcript abundance. The graphs show the results of three independent experiments. Lowercase letters indicate significant differences (two-way ANOVA, *P* < 0.05) between the expression of each gene for different groups as follows: **(A)** a, effect of the genotype (each genotype was compared to the wild type separately for roots and nodules); b, effect of the inoculation (nodules were compared with roots for each genotype); **(B)** a, effect of the age of the wild-type roots; b, effect of the inoculation for each time point.

The expression of *GSH1*, *GSHS*, and *hGSHS* was examined in 3-week-old uninoculated roots and nodules of all the genotypes. The *GSH1* level was similar between wild-type nodules and roots (Figure 5A); in contrast, *GSH1* expression was significantly upregulated in *sym40-1*, *sym33-2*, and *sym33-3* mutant nodules when compared with roots and wild-type nodules.

*GSHS* expression was significantly higher in nodules of all genotypes than in roots. *GSHS* mRNA levels were significantly higher in *sym40-1* and *sym33-3* mutant nodules than in wild-type nodules. However, *GSHS* expression in nodules of the *sym33-2* mutant, blocked at the earliest stage of development among the mutants (without bacterial release), was significantly lower than that in wild-type nodules. The highest *GSHS* expression level was observed in *sym40-1* nodules. The roots of all the genotypes, except *sym33-2*, displayed a higher level of *hGSHS* expression than the nodules. The *hGSHS* expression level was similar between *sym33-2* nodules and roots and gradually decreased in nodules in a developmental stage-dependent manner (from *sym33-3* to *sym40-1* and wild type).

The gene expression levels were quantified in 1- and 2-week-old roots and nodules of wild-type plants (Figure 5B). Compared with roots, there was a significant upregulation of *GSHS* expression and a slight downregulation of that of *hGSHS* in 1-week-old nodules. *GSHS* expression remained high in 2-week-old nodules, whereas that of *hGSHS* was significantly suppressed.

**FIGURE 6:**
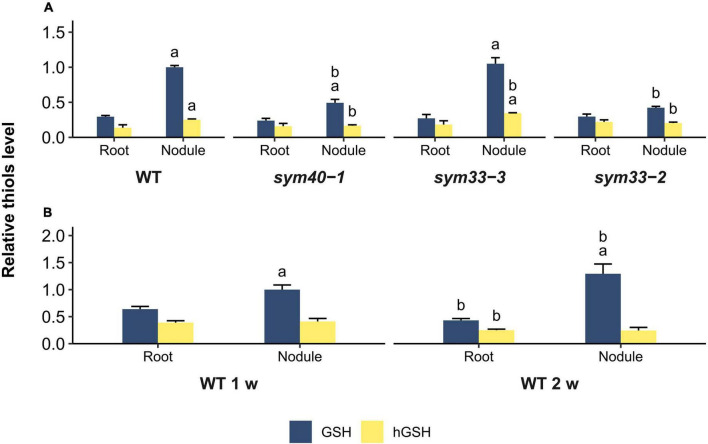
Relative thiol levels in nodules and roots of wild-type and mutant (*sym40-1*, *sym33-3*, and *sym33-2*) pea (*Pisum sativum*) plants. **(A)** Three-week-old uninoculated roots and nodules. **(B)** One- and two-week-old uninoculated roots and nodules. The values are given relative to glutathione content in **(A)** 3-week-old wild-type nodules and **(B)** 1-week-old wild-type nodules. GSH/hGSH amount in the samples were normalized relative to the GSH amount in 1- or 3-week-old wild-type nodules. The amount of thiols in nmol/g presented in Supplementary Table 2. The graphs show the results of four independent experiments. Lowercase letters indicate significant differences (two-way ANOVA, *P* < 0.05) in content of each thiol for different groups as follows: **(A)** a, effect of the inoculation for each genotype; b, effect of the genotype (each genotype was compared to the wild type); **(B)** a, effect of the inoculation for each time point; b, effect of the age for roots and nodules.

To estimate the correlation between changes in the expression of (h)GSH biosynthesis-related genes and actual thiol levels, a quantitative determination of low-molecular-weight thiols in 1-, 2-, and 3-week-old roots and nodules was undertaken (Figures 6A,B). In all genotypes, the GSH concentration and the GSH:hGSH ratio were significantly higher in nodules than in roots (Figures 6A,B and Supplementary Table 2). However, among all the nodules, those of the *sym33-2* mutant displayed the lowest GSH:hGSH ratio. In *sym33-3* nodules, GSH levels were comparable to those of wild-type, while the hGSH content was higher, leading to a decrease in the GSH:hGSH ratio. The levels of GSH and hGSH were lower in *sym40-1* nodules than in wild-type or *sym33-3* nodules despite *sym40-1* nodules exhibiting the highest *GSHS* expression levels. Nevertheless, the GSH:hGSH ratio in *sym40-1* nodules was the same as that in *sym33-3* nodules and higher than that in *sym33-2* nodules. Additionally, although *hGSHS* expression was lower in nodules than in control roots, there was no corresponding decrease in hGSH content. Indeed, compared with roots, the hGSH level was higher in wild-type and *sym33-3* nodules, and was unchanged in *sym40-1* and *sym33-2* nodules.

The GSH:hGSH ratio was highest in wild-type nodules and gradually decreased in mutant nodules in a developmental stage-dependent manner (Supplementary Table 2). However, each of the genotypes showed a specific thiol composition in nodules. The highest GSH concentration was observed in wild-type and *sym33-3* nodules and that of hGSH in *sym33-3* nodules.

To understand whether such variability in thiol content is related to the stage of nodule development at which the mutants are blocked or to metabolic changes in ineffective nodules, we also analyzed the root tissue and nodules of 1- and 2-week-old plants (Figure 6B). The GSH:hGSH ratio was similar among 1-, 2-, and 3-week-old roots (Supplementary Table 2). The GSH concentration and the GSH:hGSH ratio in nodules increased at 2 weeks post-inoculation. Among all the samples, the GSH:hGSH ratio was highest in 2-week-old wild-type nodules (Supplementary Table 2), while GSH was the main thiol identified.

These data suggested that, for the normal development of a symbiotic nodule, several conditions must be met: (i) the amount of GSH must increase in nodule tissues compared to the roots; (ii) the amount of hGSH must not exceed a certain level; and (iii) the GSH:hGSH ratio must reach a certain value. However, further research should be aimed at confirming this suggestion.

### The Effect of (h)GSH Depletion on Wild-Type and Mutant Nodule Morphology and Gene Expression

**FIGURE 7:**
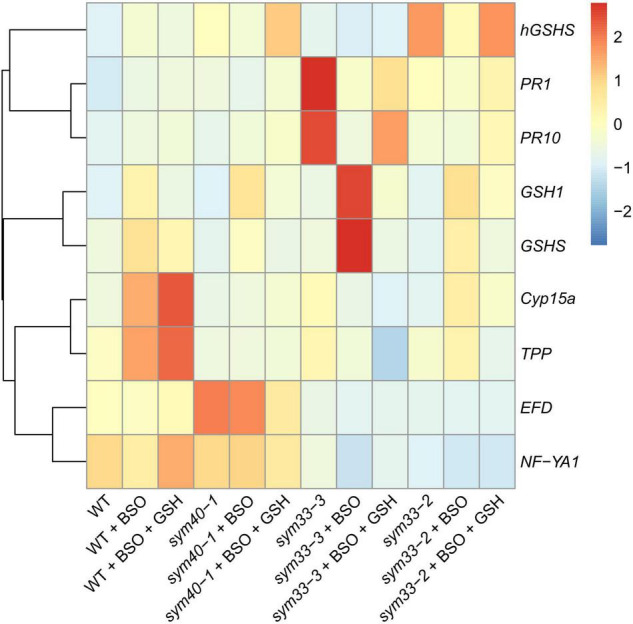
Heatmap showing relative gene expression levels in 2-week-old roots with nodules from wild-type and mutant pea (*Pisum sativum*) plants treated with L-buthionine-sulphoximine (BSO) and glutathione (GSH). Transcript levels were determined by real-time PCR and calculated using the ΔCT method with glyceraldehyde-3-phosphate dehydrogenase (*GAPC1*) serving as the reference gene. The color scale shows relative expression values for each gene after *Z*-transformation. The heatmap is based on data presented in Supplementary Table 3 (list 1). Gene expression levels were compared using one-way ANOVA, a *P*-value < 0.05 was considered significant [see also Supplementary Table 3 (list 2)].

BSO was used to induce (h)GSH deficiency and distinguish the roles of GSH and hGSH during symbiotic nodule development and defense responses. Subsequently, the effects of (h)GSH depletion on nodule development and functioning were assessed at both the morphological and transcriptional levels. For this, nodule structure and gene expression profiles—including the expression of genes associated with nodule development (*EFD*, *NF-YA1*), (h)GSH biosynthesis (*GSH1*, *GSHS*, and *hGSHS*), markers of defense responses (*PR1*, *PR10*), and nodule senescence markers (*TPP*, *Cyp15a*)—were compared among untreated, BSO-treated, and BSO+GSH-treated roots with nodules (Figure 7).

**FIGURE 8:**
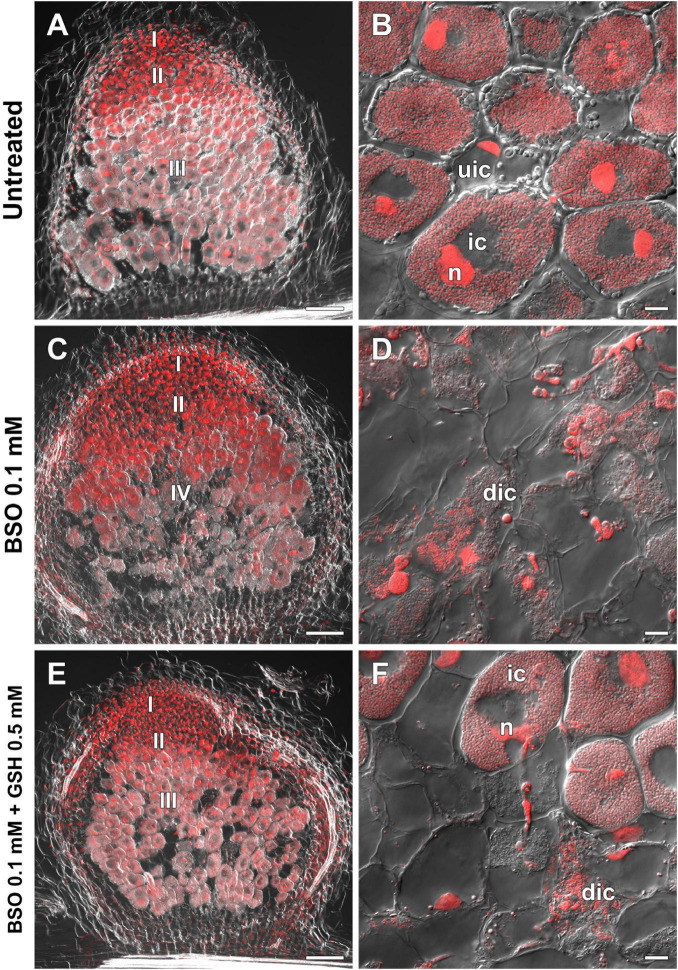
Two-week-old nodules from wild-type pea (*Pisum sativum*) plants treated with L-buthionine-sulphoximine (BSO) and glutathione (GSH). **(A,C,E)** General view; **(B,D,F)** infected cells. **(A,B)** untreated nodules; **(C,D)** nodules on plants treated with 0.1 mM BSO; **(E,F)** nodules on plants treated with 0.1 mM BSO and 0.5 mM GSH. Confocal laser scanning microscopy images of 50-μm-thick longitudinal sections. Merge of differential interference contrast and the red channel. A single optical section is presented. DNA (bacteria and nuclei) is shown in red. I, meristematic zone; II, infection zone; III, nitrogen fixation zone; IV, senescence zone; n, nucleus; ic, infected cell; uic, uninfected cell; dic, degrading infected cell. Scale bars = 100 μm **(A,C,E)** and 10 μm **(B,D,F)**.

High-performance liquid chromatography analysis showed that BSO treatment led to a 94 and 96% reduction in GSH and hGSH content, respectively, in wild-type roots with nodules (Supplementary Table 4). Nodules on untreated, wild-type plants grown without cotyledons were pink and had typical indeterminate nodule histological organization (Figures 8A,B). A 46% reduction in nodule number was observed in BSO-treated wild-type plants (Supplementary Table 4). Microscopic analysis of BSO-treated nodules revealed a premature degradation of symbiotic structures at the base and center of the nodules, indicative of early senescence (Figures 8C,D). *Cyp15a, TPP*, *PR1*, and *PR10* expression was upregulated and that of *NF-YA1* downregulated in response to (h)GSH depletion in wild-type roots with nodules (Figure 7). Compared with untreated plants, BSO+GSH-treated plants had the same number of nodules and exhibited higher levels of GSH, but not hGSH, in roots with nodules (237 and 88%, respectively) (Supplementary Table 4). This change in thiol content coincided with the upregulation of *NF-YA1*, *Cyp15a, TPP*, *PR1*, and *PR10* expression levels in BSO+GSH-treated plants relative to untreated plants (Figure 7). Nodules on the roots of BSO+GSH-treated plants did not show signs of early senescence in the nitrogen fixation zone (Figures 8E,F); however, some cells showed signs of degradation (Figure 8F).

**FIGURE 9:**
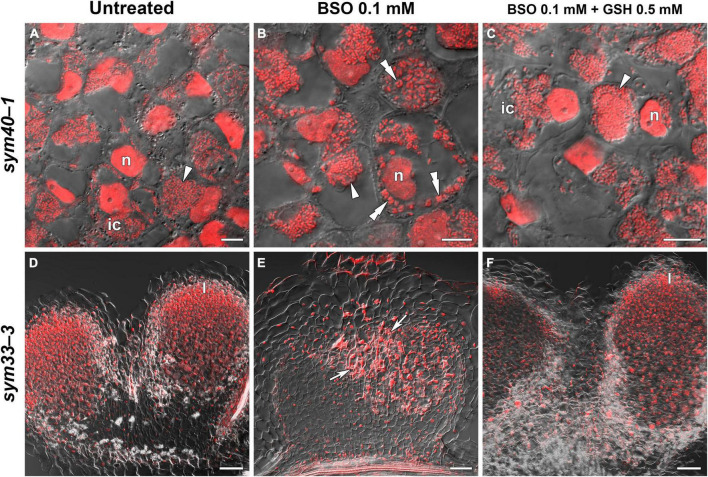
Two-week-old nodules from mutant (*sym40-1* and *sym33-3*) pea (*Pisum sativum*) plants treated with L-buthionine-sulphoximine (BSO) and glutathione (GSH). **(A–C)** Cells of *sym40-1* nodules; **(D–F)** general view of *sym33-3* nodules; **(A,D)** untreated nodules; **(B,E)** nodules on plants treated with 0.1 mM BSO; **(C,F)** nodules on plants treated with 0.1 mM BSO and 0.5 mM GSH. Confocal laser scanning microscopy images of 50-μm-thick longitudinal sections. Merge of differential interference contrast and the red channel. A single optical section is presented, DNA (bacteria and nuclei) is shown in red. I, meristematic zone; n, nucleus; ic, infected cell. Arrowheads indicate infection droplets; double arrowheads indicate abnormal symbiosomes containing several bacteroids; arrows indicate a zone of propagation of “locked” infection threads. Scale bars = 10 μm **(A–C)** and 100 μm **(D–F)**.

The GSH and hGSH content in BSO-treated *sym40-1* roots with nodules was reduced by 94 and 99%, respectively, compared with untreated *sym40-1* roots with nodules (Supplementary Table 4). The nodule number was decreased by only 26% in BSO-treated plants and was not affected by BSO+GSH treatment (Supplementary Table 4). Microscopic analysis revealed the presence of numerous symbiosomes containing several bacteroids (in 47% of infected cells in the nodule) in BSO-treated nodules of the *sym40-1* mutant (Figure 9B and Supplementary Table 5), which were not observed in untreated (Figure 9A) or BSO+GSH-treated nodules (Figure 9C). In BSO+GSH-treated *sym40-1* roots with nodules, GSH and hGSH levels were 103 and 113%, respectively, of the levels in untreated plants. No significant changes were observed in *Cyp15a, TPP*, or *PR1* expression in BSO- and BSO+GSH-treated *sym40-1* roots with nodules; however, the expression levels of *hGSHS* and *PR10* were increased and those of *NF-YA1* and *EFD* decreased only in BSO+GSH-treated *sym40-1* roots with nodules (Figure 7). Among all the genotypes, the expression of the *EFD* gene was highest in the *sym40-1* mutant (*Sym40* encodes the EFD transcription factor); however, BSO+GSH treatment led to a decrease in *EFD* expression (Figure 7).

In *sym33-3* roots with nodules, BSO treatment led to a 97 and 99% reduction in GSH and hGSH content, respectively (Supplementary Table 4). Compared with untreated plants, the nodule number was reduced by up to 98% in BSO-treated plants and up to 50% in plants treated with BSO+GSH. The development of BSO-treated *sym33-3* nodules was impaired in the early stage, likely due to meristem arrest. Moreover, the infection threads showed signs of frequent arrest and erratic orientation and formed swollen bulbs (Figure 9E), which correlated with the downregulation of *NF-YA1* expression levels (Figure 7). The same effect was observed in the infection threads of *sym33-2* nodules (data not shown). Compared with wild-type roots with nodules, *PR1* and *PR10* expression was markedly increased in untreated *sym33-3* roots with nodules; however, the expression of these genes was strongly downregulated following (h)GSH depletion (Figure 7). In BSO+GSH-treated plants, nodule development was not impaired, even though the GSH and hGSH levels were only 38% and 18% of those of untreated plants (Figures 9D,F and Supplementary Table 4). *Cyp15a* and *TPP* expression was slightly downregulated, whereas that of *PR1*, *PR10*, and *NF-YA1* was upregulated in BSO+GSH-treated *sym33-3* roots with nodules, but less than in untreated plants (Figure 7).

On average, 2 to 3 nodules formed on *sym33-2* roots following (h)GSH depletion, which allowed the use of this mutant as an “inoculated roots without nodules” variant. In *sym33-2* roots with nodules, BSO treatment led to a 96 and 98% reduction in GSH and hGSH content, respectively (Supplementary Table 4). Compared with untreated plants, the nodule number was reduced by up to 50% in BSO-treated plants; however, nodule number was not affected in plants treated with BSO+GSH. The *Cyp15a* and *TPP* transcript levels were upregulated and those of *hGSHS* downregulated in BSO-treated *sym33-2* roots with nodules relative to those in untreated plants (Figure 7). *sym33-2* plants maintained a low level of *NF-YA1* and *EFD* expression in all variants. In BSO+GSH-treated *sym33-2* roots with nodules, GSH and hGSH levels were 290 and 49%, respectively, of the levels in untreated plants. *PR10* expression was higher in BSO+GSH-treated *sym33-2* plants than in untreated or BSO-treated plants (Figure 7).

The mRNA expression levels of *GSH1* and *GSHS* were increased in BSO-treated nodule-containing roots in all the genotypes (Figure 7).

The expression of the *7RA84*, *Hsr203J*, and *Abr17* genes (markers of the defense response and oxidative stress) has been shown to be upregulated in *sym40-1* nodules (Ivanova et al., 2015). Here, we found that the expression of these genes was also upregulated in BSO-treated wild-type nodules that displayed low thiol content and signs of early senescence, as also seen in *sym40-1* nodules (Supplementary Figure 4A).

### The Effect of Exogenously Applied Glutathione on the Morphology and Gene Expression of Wild-Type and Mutant Nodules

To evaluate the influence of thiol concentrations and that of their ratio on nodule development, 0.1 mM or 1 mM GSH was provided as a supplement to the roots of wild-type and mutant plants.

**FIGURE 10:**
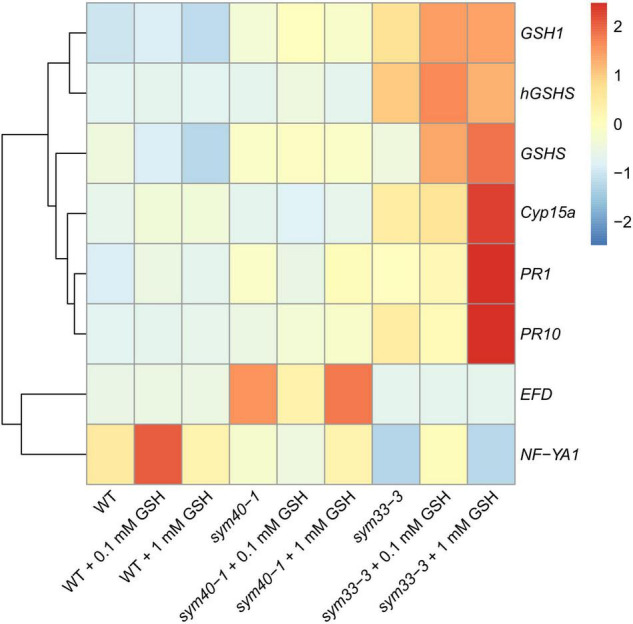
Heatmap showing relative gene expression levels in 2-week-old roots with nodules from wild-type and mutant pea (*Pisum sativum*) plants treated with 0.1 mM or 1 mM glutathione (GSH). Transcript levels were determined by real-time PCR and calculated using the ΔCT method, with glyceraldehyde-3-phosphate dehydrogenase (*GAPC1*) serving as the reference gene. The color scale shows relative expression values for each gene after *Z*-transformation. The heatmap is based on data provided in Supplementary Table 3 (list 3). Gene expression levels were compared using one-way ANOVA, a *P*-value < 0.05 was considered significant [see also Supplementary Table 3 (list 4)].

The treatment of wild-type plants with 0.1 mM GSH for 2 weeks led to a twofold increase in GSH content in roots with nodules, but did not affect the hGSH level (Supplementary Table 6). Treatment with 0.1 mM GSH induced the expression of nodule development-related genes (*EFD* and *NF-YA1*) in wild-type plants (Figure 10). Meanwhile, treatment with 1 mM GSH elicited a threefold increase in the levels of both thiols (Supplementary Table 6). No prominent morphological differences between GSH-treated and untreated wild-type nodules were observed (data not shown). The expression levels of (h)GSH biosynthesis-related genes, defense response genes (*PR1* and *PR10*), and that of the nodule senescence marker gene *Cyp15a* were not significantly changed in roots with nodules of GSH-treated wild-type plants (Figure 10).

**FIGURE 11:**
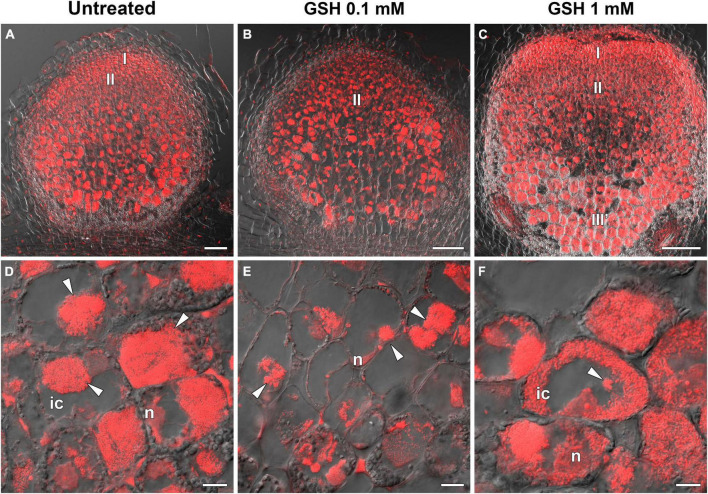
Two-week-old nodules from mutant *sym40-1* pea (*Pisum sativum*) plants treated with 0.1 mM or 1 mM glutathione (GSH). **(A–C)** General view of *sym40-1* nodules; **(D–F)** infected cells of *sym40-1* nodules; **(A,D)** untreated nodules; **(B,E)** nodules on plants treated with 0.1 mM GSH; **(C,F)** nodules on plants treated with 1 mM GSH. Confocal laser scanning microscopy images of 50-μm-thick longitudinal sections. Merge of differential interference contrast and the red channel. A single optical section is presented, DNA (bacteria and nuclei) is shown in red. I, meristematic zone; II, infection zone; III′, zone corresponding to the nitrogen fixiation zone of wild-type nodule; n, nucleus; ic, infected cell. Arrowheads indicate infection droplets. Scale bars = 100 μm **(A–C)** and 10 μm **(D–F)**.

In the *sym40-1* mutant, treatment with 0.1 mM GSH did not alter the GSH level and led to a 25% increase in that of hGSH in roots with nodules (Supplementary Table 6). This treatment led to a reduction in the size of hypertrophied infection droplets and the number of cells with bacterial release (Figures 11A,B,D,E), as well as a decrease in the expression levels of the *EFD*, *Cyp15a*, and *PR1* genes; in contrast, the expression of *PR10* and *hGSHS* was upregulated in *sym40-1* roots with nodules (Figure 10).

Similar to that seen in the wild type, treatment with 1 mM GSH almost tripled the content of both thiols (Supplementary Table 6). Treating the roots of *sym40-1* mutants with 1 mM GSH also led to a reduction in the size of hypertrophied infection droplets as well as an increase in the number of infected cells that resembled infected cells in wild-type nodules (Figures 11C,F). Only the expression level of *PR10* was upregulated in roots with nodules of 1 mM GSH-treated *sym40-1* mutant plants compared with untreated *sym40-1* plants (Figure 10).

**FIGURE 12:**
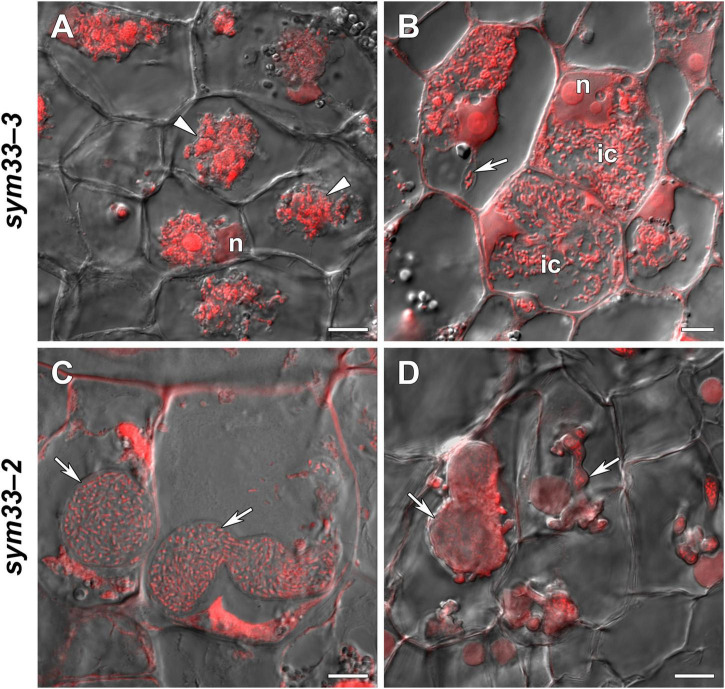
Two-week-old nodules from mutant (*sym33-3* and *sym33-2*) pea (*Pisum sativum*) plants treated with 1 mM glutathione (GSH). **(A,B)** *sym33-3* and **(C,D)** *sym33-2*. Confocal laser scanning microscopy images of 50-μm-thick longitudinal sections. Merge of differential interference contrast and the red channel. A single optical section is presented. DNA (bacteria and nuclei) is presented in red. n, nucleus; ic, infected cell. Arrows indicate infection threads; arrowheads indicate infection droplets. Scale bar = 10 μm.

*sym33-3* mutant roots with nodules displayed a 25% increase in GSH levels and a 35% decrease in the hGSH pool after treatment with 0.1 mM GSH; after treatment with 1 mM GSH, there was a 1.5-fold increase in GSH levels and a twofold increase in those of hGSH (Supplementary Table 6). Treating *sym33-3* mutants with GSH led to the formation of structures resembling infection droplets in many nodule cells, with some droplets displaying hypertrophy, especially after 1 mM GSH treatment (Figure 12A); bacterial release occurred in some cells but the released bacteria were disorganized (Figure 12B). Treating the roots of *sym33-2* mutants with 1 mM GSH did not promote infection events, but led to the overgrowth and swelling of infection threads (Figures 12C,D). Bulbs that formed on the infection threads sometimes had different protrusions (Figure 12D). No visible changes in the histological structure of *sym33-2* nodules were observed between GSH-treated (0.1 mM) and untreated plants (data not shown). The expression levels of (h)GSH biosynthesis-related genes were upregulated in *sym33-3* plants treated either with both concentrations of GSH or only with 0.1 mM GSH in the case of *hGSHS* expression (Figure 10). Treatment with 0.1 mM GSH induced the expression of the *EFD* and *NF-YA1*genes in *sym33-3* roots with nodules. Treatment with 1 mM GSH led to the upregulation of *PR1*, *PR10*, and *Cyp15a* in *sym33-3* mutants (Figure 10).

These data further confirmed the dual role of (h)GSH in the regulation of nodule development and defense responses in the pea.

The expression levels of the *7RA84*, *Hsr203J*, and *Abr17* genes (markers of defense responses and oxidative stress) were also examined. In *sym40-1* nodules treated with 0.1 mM GSH, the expression of *Abr17* and *7RA84* was downregulated (Supplementary Figure 4B).

## Discussion

### The Localization of Glutathione Was Indicative of Its Important Role in Nodule Meristem Formation, Persistence of Nitrogen-Fixing Cells, Nitrogen Fixation in Bacteroids, and Stress Responses in Rhizobia

In this study, in wild-type nodules, labeling of GSH was observed in the meristem (Figures 1D–F), which correlated with the spatial expression pattern of the *MtGSH1* gene in *M. truncatula* nodules (El Msehli et al., 2011). Labeling of GSH was also observed in nuclei of infected cells from the infection zone (Figures 1G–I), but not the nitrogen fixation zone (Figures 1J–L). Labeling was strongest in symbiosomes with mature bacteroids from cells in the nitrogen fixation zone (Figures 1J–L, 5B) and weakest in senescent bacteroids (Figure 4C and Table 2). It was not possible to determine whether GSH in nodules was of plant or bacterial origin. The significance of bacterial GSH has been demonstrated using different GSH-deficient *Rhizobium* mutants (Muglia et al., 2008; Taté et al., 2012). A recent study showed that GSH deficiency in *Sinorhizobium meliloti* 2011 (*gshB*) did not affect bacteroid differentiation, but instead induced early nodule senescence (Yang et al., 2020), indicating that GSH content in bacteroids is essential for their proper functioning.

In a previous study, using an antibody that recognizes both the reduced and oxidized forms of GSH, the strongest signal was observed in bacteroids in mature pea nodules; however, labeling was markedly reduced in senescent nodules (Matamoros et al., 2013). In addition, the amount of label was higher in the cytoplasm and nuclei of mature infected cells than in young ones. In contrast, we found that the highest levels of cytoplasmic and nuclear labeling were associated with young infected cells. That the antibody used in our study only recognized the reduced form of GSH implied that the redox state in the cytoplasm and nuclei of young infected cells was more reduced compared with that in mature infected cells. It is known that GSH is required for cell division in the root apex (Vernoux et al., 2000) and that GSH levels regulate the G1-to-S-phase cell-cycle transition (Diaz-Vivancos et al., 2010a,b), suggesting that GSH can stimulate meristematic activity and, apparently, repetitive rounds of endoreduplication in infected nodule cells.

In wild-type nodules, the amount of GSH label in bacteria in infection threads was low (Supplementary Figure 3A and Table 2), with the most intensive labeling being observed in bacteria in some “locked” infection threads of *sym33-3* mutants (Figures 3D–F) and juvenile bacteroids of *sym33-3* and *sym40-1* mutants (Figures 2D–F, 3J–L, 4D). These effects can be explained by the activation of strong defense responses in these mutants, including the suberinization of infection thread walls in *sym33-3* nodules (Ivanova et al., 2015), and hydrogen peroxide accumulation around juvenile bacteroids in *sym40-1* nodules (Tsyganova et al., 2009). This suggests that rhizobia use GSH to mitigate the stress induced by the activation of plant defenses in these mutants.

### Nodules of Different Genotypes Showed Varied Thiol Composition

Uninoculated roots and wild-type nodules were compared to identify changes in the expression pattern of (h)GSH biosynthesis genes related to symbiotic interactions. Differences between wild-type and mutant nodules could be indicative of the involvement of (h)GSH at different stages of nodule development or in defense responses associated with ineffective symbiosis.

Except for *sym33-2*, *GSHS* expression was significantly upregulated, and that of *hGSHS* downregulated in nodules of all genotypes and ages compared with that in uninoculated roots. This suggests that bacterial release is necessary for a *hGSHS*-to-*GSHS* switch in gene expression in nodule tissue, which is a requirement for nodule development. Meanwhile, *GSH1*, *GSHS*, and *hGSHS* expression was higher in *sym40-1* and *sym33-3* mutant nodules than in wild-type nodules (Figure 5A), suggestive of a change in thiol metabolism in these mutants associated with defense responses (Ivanova et al., 2015; Tsyganova et al., 2019b).

In the present study, we found that *GSHS* expression was higher, and the level of GSH lower, in *sym40-1* mutant nodules than in wild-type or *sym33-3* nodules (Figures 5A, 6A). These changes in GSH metabolism could be triggered by oxidative stress and hydrogen peroxide accumulation, effects that are observed in nodules of *sym40-1* mutants and are associated with the perception of nodule bacteria as pathogens rather than microsymbionts (Tsyganova et al., 2009; Ivanova et al., 2015). Indeed, treatment with jasmonic acid, a stress-related hormone, was reported to increase the *GSH1* and *GSHS* transcript levels in *Arabidopsis thaliana* without affecting the GSH level (Xiang and Oliver, 1998). Additionally, in *A. thaliana pad2-1* mutants, the expression of GSH biosynthesis-related genes was increased in response to attack by the pathogen *Phytophthora brassicae* (Parisy et al., 2007).

Glutathione was the main thiol in all analyzed samples. Nevertheless, the GSH:hGSH ratio was always higher in nodules with bacterial release than in control roots or *sym33-2* nodules without bacterial release. For wild-type nodules, the GSH:hGSH ratio was lowest in 1-week-old nodules, intermediate in 3-week-old nodules, and highest in 2-week-old nodules (Supplementary Table 2). We suggest that the maximal GSH:hGSH ratio correlates with the highest level of meristem activity, which occurs in 2-week-old pea nodules (Voroshilova et al., 2009). In turn, the decrease in the GSH:hGSH ratio observed in 3-week-old pea nodules correlates with a decline in meristem activity and an increase in the number of fully differentiated cells.

Taken together, these findings indicate that the GSH:hGSH ratio is an important factor in indeterminate nodule development and functioning. A high GSH:hGSH ratio is associated with bacterial release, the functioning of the nodule meristem, and likely also the differentiation of nitrogen-fixing cells. Indeed, in *M*. *sativa*, both GSH and hGSH are present in all organs; however, their ratio varies across organs, being highest in root meristems and lowest in mature leaves and the root elongation zone. GSH was associated with cell-cycle activation in cell suspension cultures, whereas hGSH was associated with differentiated cells (Pasternak et al., 2014).

Although all mutant nodules exhibited a distinct thiol composition, the GSH:hGSH ratio correlated with the extent of infection (Supplementary Table 2). Additionally, the (h)GSH content was highest in *sym33-3* nodules and lowest in *sym40-1* nodules (Figure 6A). This suggests that changes in the contents of both thiols in ineffective symbiotic nodules may depend on the stage at which symbiosis is blocked and the defense response activated. In the *sym33-3* mutant, where most of the infection threads is “locked,” thus inhibiting bacterial release, the accumulation of both GSH and hGSH is likely to be important for “disease” resistance, as has been suggested for GSH in *A. thaliana* (Parisy et al., 2007). (h)GSH accumulation coincided with the upregulation of *PR1*, *PR10* (Figures 7, 10), *7RA84* (peroxidase), and *Hsr203J* (hypersensitivity response marker) expression in *sym33-3* mutant nodules relative to wild-type nodules, as well as manifestations of other defense responses, such as suberinization, increased unesterified pectin deposition in infection threads walls, and increased cell wall material deposition around the vacuole (Ivanova et al., 2015; Tsyganova et al., 2019b). GSH was shown to promote the accumulation of pathogenesis-related (PR) proteins and the expression of defense-related genes (Ball et al., 2004; Gomez et al., 2004). As in the *sym33-3* mutant, *hGSHS* expression was also found to be upregulated in *M*. *truncatula nad1* mutant nodules compared with wild-type nodules. *nad1* mutant nodules are blocked in development immediately after bacterial release and are characterized by phenolic compound accumulation, increased hydrogen peroxide levels, and activation of defense-related genes (Wang et al., 2016).

Nodule development in *sym40-1* plants stops after the differentiation of juvenile bacteroids (Tsyganov et al., 1998). Decreased (h)GSH content in nodules may also be associated with plant defense responses triggered by the perception of nodule bacteria as pathogens. Changes in (h)GSH content have been shown to affect defense responses during biotic stress in some legumes (Baldacci-Cresp et al., 2012; Chen et al., 2020). (h)GSH is essential for the growth of parasitic nematode worms that infect plant roots and force the differentiation of root cells into giant cells, forming galls. (h)GSH metabolism differs between galls and uninfected roots. (h)GSH-depleted *M*. *truncatula* plants are less affected by infection of root-knot nematodes (Baldacci-Cresp et al., 2012). Hypersensitive response-like cell death and hydrogen peroxide production around the area of nematode infection have been observed in (h)GSH-depleted *Glycine max* roots (Chen et al., 2020), resembling hydrogen peroxide accumulation in mutant *sym40-1* nodules (Tsyganova et al., 2009).

Accordingly, we conclude that thiol composition in ineffective nodules, as well as the spectrum of defense responses, depends on the stage at which symbiosis is blocked.

### (h)GSH Depletion Negatively Influences Nodule Development, While Exogenous Glutathione Application Promotes Infection and Defense Responses in *sym33-3* Mutants and the Maturation of Infected Cells in *sym40-1* Mutants

The expression levels of *GSH1*, *GSHS*, *Cyp15a, TPP*, *PR1*, and *PR10* were upregulated in (h)GSH-depleted wild-type roots with nodules (Figure 7); however, no significant change in the expression of these genes was observed in roots with nodules of GSH-treated wild-type plants (Figure 10). This indicates that the expression of these genes is responsive to reduced (h)GSH concentrations. BSO treatment promoted an early induction of the senescence zone in wild-type nodules (Figures 8C,D) concomitant with an increase in the expression of the senescence-associated genes *Cyp15a* and *TPP* (Serova et al., 2017; Figure 7). This demonstrates that (h)GSH is crucial for the functioning of nitrogen-fixing cells in wild-type pea nodules. A reduction in thiol content during nodule senescence has been reported in *G. max* (Evans et al., 1999), pea (Groten et al., 2005), and *Phaseolus vulgaris* (Loscos et al., 2008).

BSO-treated *sym40-1* nodules formed numerous symbiosomes containing several bacteroids surrounded by a common symbiosome membrane (Figure 9B). A similar phenotype was previously described for some senescent cells from *sym40-1* nodules (Tsyganov et al., 1998). The formation of symbiosomes containing several bacteroids has been reported to result from mutations in different pea symbiotic genes (Novák et al., 1995; Borisov et al., 1997; Morzhina et al., 2000) as well as boron deficiency (Bolaños et al., 2001), cadmium exposure (Tsyganova et al., 2019c; Tsyganov et al., 2020), or tetramethylthiuram disulfide treatment (Gorshkov et al., 2020). In boron-deficient plants and *sym31* mutant, the formation of symbiosomes containing several bacteroids was associated with mistargeting of the lectin-like glycoprotein NLEC-1 (Dahiya et al., 1998; Bolaños et al., 2001). Modified NLEC-1 cannot bind to the cell surface of *Rhizobium leguminosarum* 3841 (Bolaños et al., 2004), leading to defective symbiosome division. Analyzing NLEC-1 distribution in BSO-treated *sym40-1* nodules merits further investigation.

In (h)GSH-depleted *sym33-3* nodules, meristem development was blocked, and infection thread growth was interrupted (Figure 9E). These morphological changes coincided with the downregulation of *NF-YA1* expression (Figure 7). *NF-YA1* encodes a CCAAT box-binding transcription factor that controls infection and is required for nodule meristem persistence (Combier et al., 2006). The *Mtnf-ya1-1* mutant is characterized by bulbous and erratic infection thread growth that can be explained by altered infection thread cell wall integrity (Laporte et al., 2014). Combined, these observations suggest that GSH is involved not only in meristem persistence and functioning, but also in regulating infection thread growth.

A reduction in (h)GSH synthesis significantly decreased the nodule number in all genotypes tested (Supplementary Table 4). This agreed with that observed for *M*. *truncatula*, where the inhibition of (h)GSH synthesis was shown to reduce the number of nascent nodules and lateral roots and the expression of the early nodulin genes *ENOD12* and *ENOD40* without affecting the number of infection events (Frendo et al., 2005). These observations indicate that the decrease in nodule number observed in BSO-treated plants of all genotypes could be due, at least partly, to impaired meristem formation in root tissue. Interestingly, the nodule number did not differ between untreated and BSO+GSH-treated plants for all genotypes, except *sym33-3* (Supplementary Table 4). This implies that additional factors may regulate nodule number in *sym33-3* plants. For instance, the synthesis of salicylic acid (SA), a negative regulator of nodulation (Sun et al., 2006; Ding et al., 2008; Tsyganova and Tsyganov, 2018), could be increased in nodules of the *sym33-3* mutant with the manifestation of defense responses. Indirect evidence for this possibility was provided by the high expression levels of the SA-induced gene *PR1* in untreated and BSO+GSH-treated *sym33-3* plants relative to other genotypes (Figure 7). That the GSH and hGSH levels in the roots with nodules of BSO+GSH-treated *sym33-3* plants amounted to only 38 and 18% of those of untreated plants indicates that a minimal GSH level is essential for meristem functioning; however, the levels recorded in this mutant were insufficient to restore nodule numbers.

The treatment of wild-type plants with GSH did not elicit prominent phenotypic manifestations in nodule organization; however, 0.1 mM GSH treatment led to a significant increase in the expression of the *EFD* and *NF-YA1* genes (Figure 10).

s*ym40-1* mutant plants treated with 0.1 mM GSH were characterized by increased levels of *hGSHS* expression, the downregulation of *EFD* expression, and a reduction of hGSH levels in roots with nodules, in which a decrease in the size of infection droplets was observed. The expression of *hGSHS* was reported to be decreased in *L. japonicus* nodules treated with cytokinins (Clemente et al., 2012), indicating that these hormones can mediate the association between *hGSHS* and *EFD* expression. Treating s*ym40-1* mutants with 1 mM GSH resulted in an improvement in the maturation of infected cells resembling infected cells in wild-type nodules. This observation indicates that, at a certain level, GSH application can partly reverse the mutant phenotype.

Infection seemed to be more prominent in nodules of *sym33-3* mutants treated with 1 mM GSH than in untreated plants (Figure 12A); however, infection was accompanied by an increase in the expression of *Cyp15a* and PR-related genes (Figure 10) and disorganization of released bacteria (Figure 12B). Infection stimulation in these nodules may enhance defense responses that subsequently lead to the degradation of infected cells.

Finally, our experiments with (h)GSH depletion and exogenous GSH treatment allowed us to conclude that (i) both the GSH concentration and the GSH:hGSH ratio play an important role in defense responses and senescence in symbiotic nodules at least partly through the regulation of *Cyp15a, TPP*, *PR1*, and *PR10* gene expression; (ii) GSH exerts a concentration-dependent effect on *NF-YA1* gene expression, which was downregulated in (h)GSH-depleted wild-type and *sym33-3* roots with nodules and upregulated in both genotypes following treatment with the low (0.1 mM), but not high (1 mM) concentration of GSH; and (iii) *EFD* expression is upregulated in samples where the GSH:hGSH ratio changed and the GSH content reached levels twofold higher than those of hGSH (WT + 0.1 mM GSH and *sym33-3* + 0.1 mM GSH treatments), but was downregulated in the cases where hGSH content was higher than that of GSH (*sym40-1* + 0.1 mM GSH treatment) (Supplementary Table 6). These data, together with the results of the microscopic analysis of *sym40-1* nodules treated with 0.1 mM GSH, where a decrease in the size of hypertrophied infection droplets was observed, as well as the fact that the highest hGSH content was detected in *sym33-3* nodules, where infection threads rarely formed infection droplets, suggest that hGSH may play a role in infection droplet formation.

**FIGURE 13:**
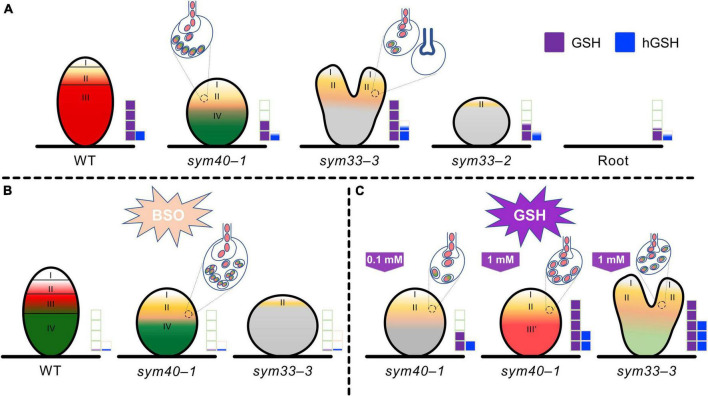
Changes in glutathione (GSH) and homoglutathione (hGSH) content affect the development of infection threads, infection droplets, and symbiosomes in pea (*Pisum sativum*) symbiotic nodule. **(A)** The effect of changes in GSH and hGSH content was analyzed in wild-type (WT) nodules and nodules of mutants defective in infection thread, infection droplet, and symbiosome development. *sym40-1* mutant nodules are characterized by formation of hypertrophied infection droplets, abnormal bacteroids, an early senescence. The *sym33-3* mutant has a leaky phenotype. Most cells in white nodules are filled with “locked” infection threads, but bacterial release occurs in some cells of some nodules. The *sym33-2* mutant manifests a severe phenotype characterized by formation of a highly branched network of “locked” infection threads arrested in the root outer cortex. Each genotype showed a specific thiol composition in nodules. In all genotypes, the GSH concentration was significantly higher in nodules than in roots. Among all the nodules, those of the *sym33-2* and *sym40-1* mutant displayed the lowest GSH and hGSH concentration. In *sym33-3* nodules, GSH levels were comparable to those of wild-type, while the hGSH content was higher. Compared with roots, the hGSH level was higher in wild-type and *sym33-3* nodules, and was unchanged in *sym40-1* and *sym33-2* nodules. **(B)** The application of a (h)GSH biosynthesis inhibitor L-buthionine-sulfoximine (BSO) induced (h)GSH deficiency in the roots with nodules. BSO treatment promoted a premature degradation of symbiotic structures at the base and center of the wild-type nodules, indicative of early senescence. The presence of numerous symbiosomes containing several bacteroids was shown in the infected cells of BSO-treated nodules of the *sym40-1* mutant. In (h)GSH-depleted *sym33-3* nodules meristem development was blocked and infection thread growth was interrupted. **(C)** An increase in hGSH levels in *sym40-1* nodules compared with untreated plants resulting from GSH treatment (0.1 mM) manifested as a restriction of infection and led to a reduction in the size of hypertrophied infection droplets and the number of cells with bacterial release. An increase in the levels of both thiols following GSH treatment (1 mM) nodules led to formation of zone resembling nitrogen fixation zone in wild-type nodule. Treating *sym33-3* mutants with GSH led to prominent infection and formation of the structures resembling infection droplets in many nodule cells. However, infection was accompanied by disorganization of released bacteria and enhanced defense responses. Zones of nodule are designated by Roman numerals and colors: I – meristematic zone (white), II – infection zone (ocher), III – nitrogen fixation zone (red), and III′ – zone corresponding to nitrogen fixation zone in wild-type nodule (reddish), and IV – senescence zone (green). Gray color – nodule parenchyma. Bacteria and bacteroids without signs of degradation are given in red. Degraded bacteroids are given in green. The levels of glutathione and homoglutathione in each genotype are represented by violet and blue squares, respectively.

In conclusion, the GSH and hGSH contents, as well as their ratio, are crucial for the selection between symbiosis- and defense-related responses during pea root rhizobial infection (Figure 13). A higher GSH to hGSH ratio is necessary for the development of symbiosis and bacterial release (Figure 13). In pea–rhizobial symbiosis, GSH is involved in meristematic cell activity, and likely also endoreduplication in infected cells, infection thread growth, the maintenance of nitrogen fixation in bacteroids, and the persistence of nitrogen-fixing cells (Figure 13). In turn, hGSH may be involved in infection droplet formation (Figure 13). Finally, the symbiosis- and defense-related functions of GSH and hGSH must be finely balanced. However, additional studies are needed to further clarify the differential roles of GSH and hGSH in the development and function of symbiotic nodules.

## Data Availability Statement

All datasets generated for this study are included in the manuscript and/or the Supplementary Material.

## Author Contributions

KI performed the experiments. AT performed the immuno-electron microscopy studies. EC and IR performed the HPLC–HRMS analysis. OK helped with the HPLC–HRMS analysis. PK contributed to the statistical analysis and figure preparation. KI and VT wrote the manuscript, with contributions from AT. IT supervised the project. VT obtained the grant that supported the research. All authors contributed to the article and approved the submitted version.

## Conflict of Interest

The authors declare that the research was conducted in the absence of any commercial or financial relationships that could be construed as a potential conflict of interest.

## Publisher’s Note

All claims expressed in this article are solely those of the authors and do not necessarily represent those of their affiliated organizations, or those of the publisher, the editors and the reviewers. Any product that may be evaluated in this article, or claim that may be made by its manufacturer, is not guaranteed or endorsed by the publisher.

## Footnotes

1
http://www.R-project.org/
