# Supplementary material for: The Regulation of Pea (Pisum sativum L.) Symbiotic Nodule Infection and Defense Responses by Glutathione, Homoglutathione, and Their Ratio
Source: Front Plant Sci. 2022 Mar 30;13:843565. doi: 10.3389/fpls.2022.843565 (PMC9006610; doi:10.3389/fpls.2022.843565)
Supplement: Supplementary file 1 [file Data_Sheet_1.pdf]

Supplementary Material

1.1 Supplementary Figures

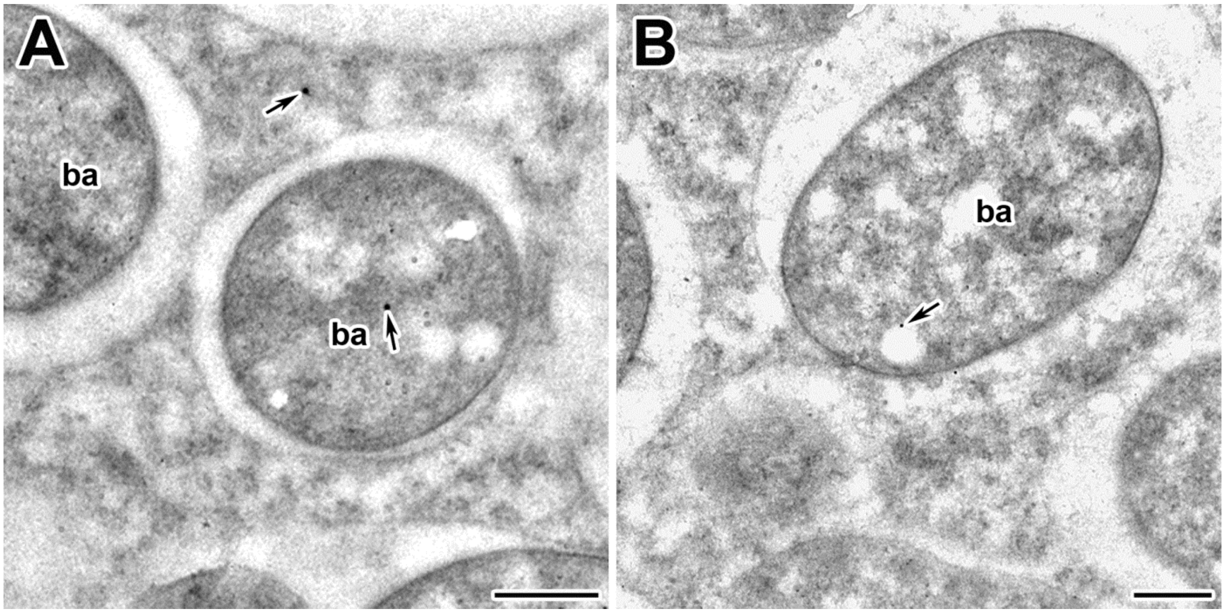

Supplementary Figure 1. Transmission electron micrographs of cells from 2-week-old pea (*Pisum sativum*) nodules treated as negative control. Gold particle labeling was sporadic or absent when cells (**A**) were treated with unspecific secondary antibody conjugated to 10-nm diameter colloidal gold and (**B**) after the omission of the primary antibody. ba, bacteroid; arrows indicate gold particles. Scale bar = 200 nm.

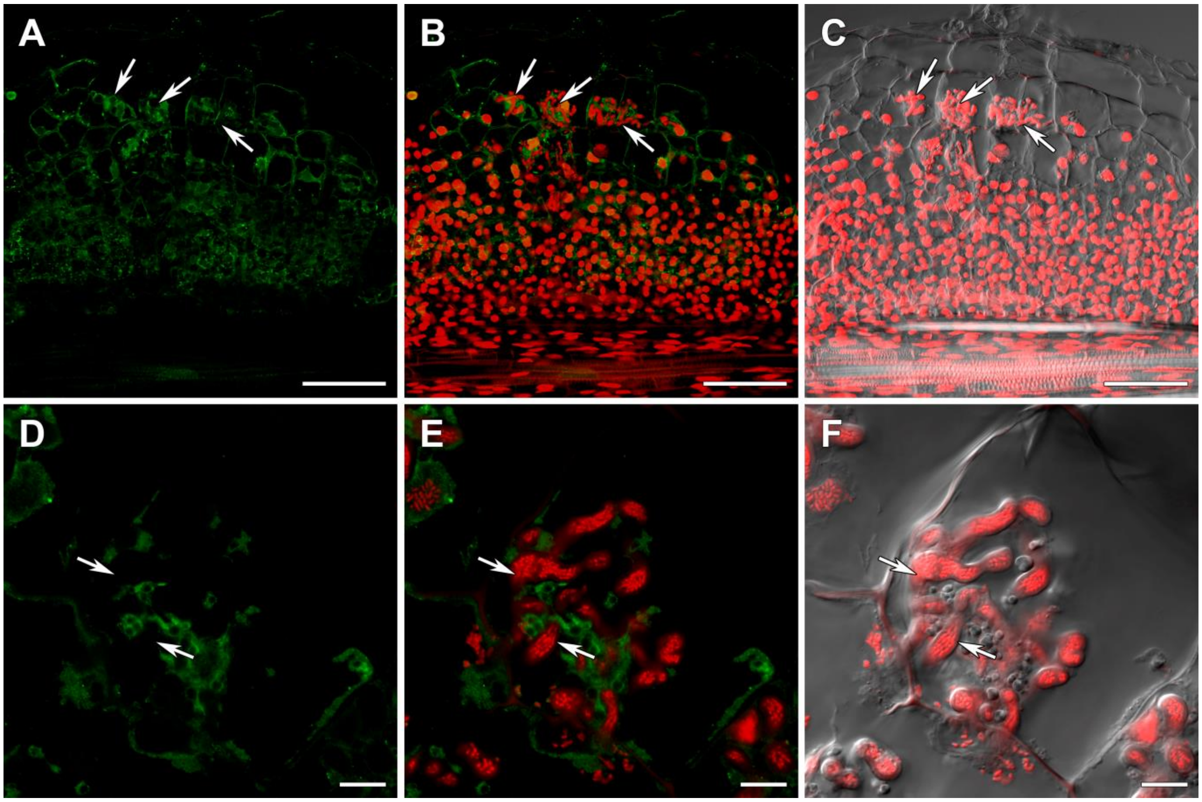

Supplementary Figure 2. Immunolocalization of glutathione in 3-week-old nodules of the pea (*Pisum sativum*) *sym33-2* mutant using a rabbit anti-glutathione (reduced) antibody. **(A–C)** General view; **(D–F)** cells with ‘locked’ infection threads. **(A, D)** Green channel; **(B, E)** merge of the green and red channels; **(C, F)** merge of differential interference contrast and the red channel. Confocal laser scanning microscopy images of 50-µm-thick longitudinal sections. A single optical section is presented, glutathione in green and DNA (bacteria and nuclei) in red. Arrows indicate infection threads. Scale bars: 100 µm **(A–C)** and 10 µm **(D–F)**.

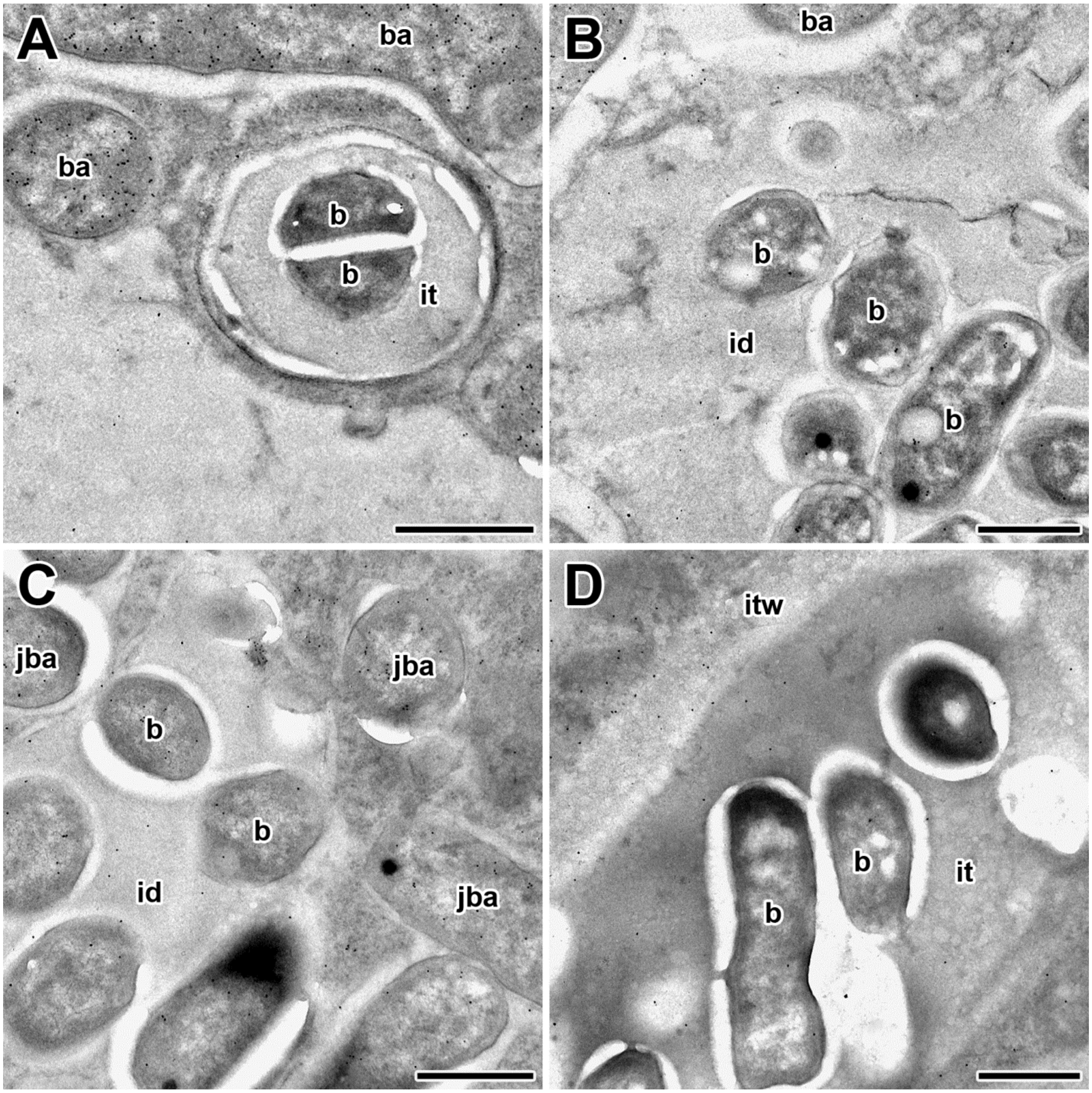

Supplementary Figure 3. Immunogold localization of glutathione in infection threads and infection droplets from 2-week-old wild-type (**A, B**) and mutant *sym40-1* (**C**) and *sym33-3* (**D**) pea (*Pisum sativum*) nodules. Secondary goat anti-rat IgG MAb conjugated to 10-nm diameter colloidal gold was used. it, infection thread; itw, infection thread wall; id, infection droplet; b, bacterium; ba, bacteroid; jba, juvenile bacteroid. (**A, D**) Infection threads. (**B, C**) Infection droplets. Scale bar = 500 nm.

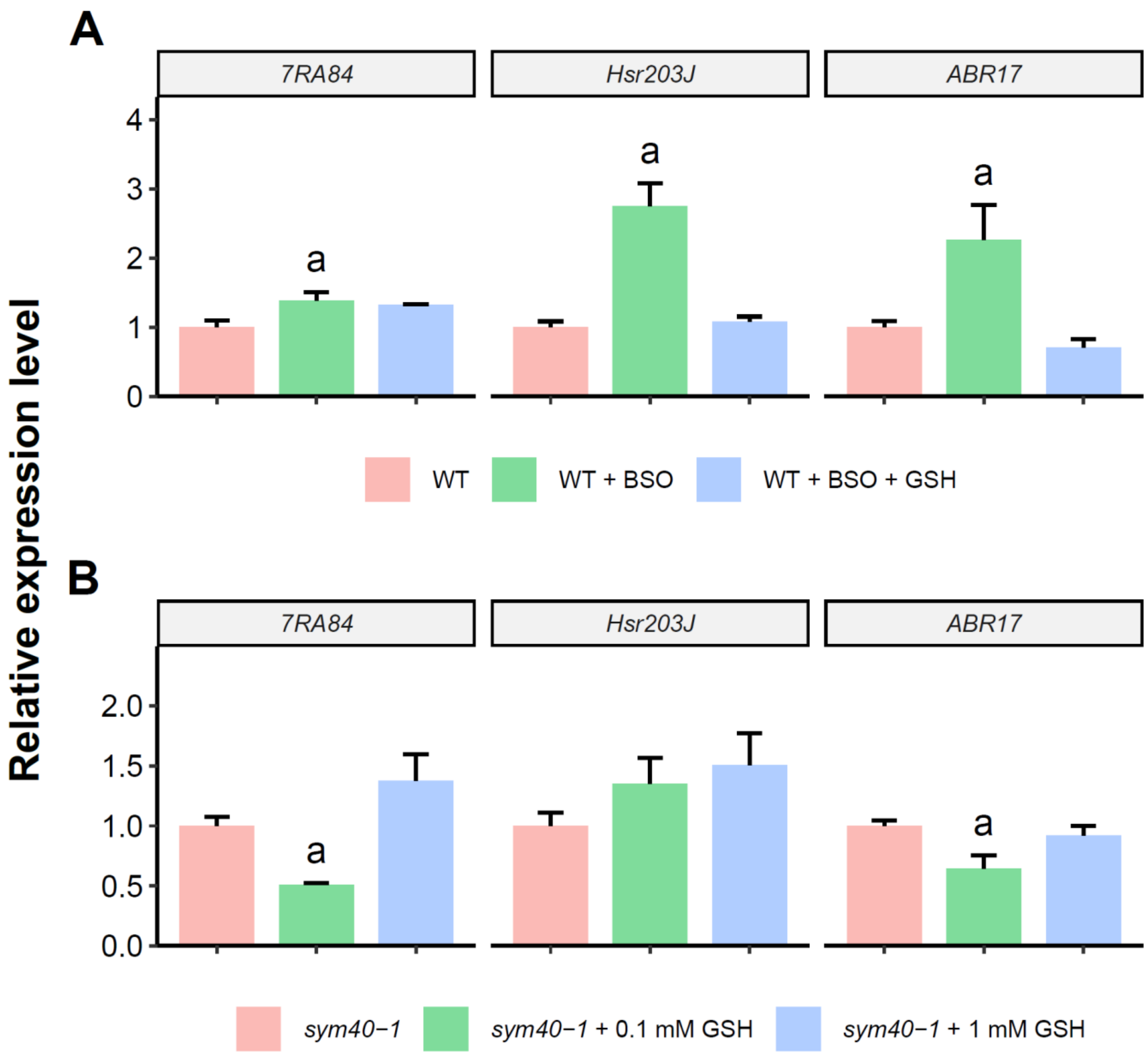

Supplementary Figure 4. The relative expression levels of the *7RA84*, *Hsr203J*, and *Abr17* genes (markers of defense responses and oxidative stress) in 2-week-old roots with nodules from wild-type (WT) pea (*Pisum sativum*) plants treated with buthionine sulphoximine (BSO) and glutathione (GSH) **(A)** and *sym40-1* mutant pea plants treated with glutathione (GSH) **(B)**. Transcript levels were determined by real-time PCR and analyzed using the $2^{-\Delta\Delta Ct}$ method, with glyceraldehyde-3-phosphate dehydrogenase (*PsGAPC1*) serving as the reference gene. Untreated wild-type roots with nodules **(A)** and untreated *sym40-1* roots with nodules **(B)** were used as calibrators for the calculation of relative transcript abundance. The graphs show the results of three independent experiments. The letter ‘a’ indicates significant differences between treated and untreated plants for each gene (one-way ANOVA, $P < 0.05$).

2 Supplementary Tables

Supplementary Table 1. List of primers used for real-time PCR

Gene	Product	Primer sequence (5'- 3')
GAPC1	Glyceraldehyde-3-phosphate dehydrogenase	F - AAGAACGACGAACTCACCG<br>R - TTGGCACCACCCTTCAAATG
GSH1	γ-Glutamylcysteine synthetase	F - CTCCTCCGCCGCATAACTTC<br>R - GGCGAGATAATCGATGAGATCCTG
GSHS	Glutathione synthetase	F - GCCGCTGATTTTCGTTCCACTA<br>R - CGACGTCGACGGTTTGTTTACC
hGSHS	Homoglutathione synthetase	F - GTTGTTGATTGATGGCTTGCATG<br>R - GCGCCAAAATCCATTGTGAA
Cyp15a	Cysteine protease 15a	F - GTAGCTGCAGCTCAATCCAACC<br>R - CATCACCACAGTAACAGCAAGACA
TPP	Thiol protease	F - TGAATCCCCCTAAGCCTGCT<br>R - GCCGGAGTTCGTTTGAATGAC
EFD	Transcription factor belongs to the ethylene response factor group V	F - ATTGGGGTTCTTGGGTCTCCGA<br>R - GATTGTGGTCCATTTGGATTGTAT
NF-YA1	CCAAT box-binding transcription factor	F - CTGACGACGGACCAACTTAC<br>R - GCTTTTCCCGTTCCCATTTG
PR1	Disease resistance protein	F - GGGGTCCATATGGTGAGAAC<br>R - TAATAACCAGGTGGATCATAGTTACA
PR10	Disease resistance protein PR10.1 (DRR49a), Putative RNAse	F - GCCGGAACCATCAAGAAACT<br>R - GCCTTGAAAAGACCATCACCC
7RA84	Peroxidase	F - TGTTTGAATCAGATGCTGCATTG<br>R - CATTTGATTGAAGATGTTGTGCAA
Hsr203J	Marker of hypersensitive reaction	F - TGTTTGAATCAGATGCTGCATTG<br>R - CATTTGATTGAAGATGTTGTGCAA
ABR17	ABA-responsive protein, PR10.4	F - TGGGTGTCTTTGTTTTTGATGATGA<br>R - TATGGCCTTGATAAGTCCAGTTCCT

Supplementary Table 2. Thiol content and GSH : hGSH ratio in 1-, 2-, and 3-week-old pea nodules and uninoculated (control) root

	GSH	hGSH	GSH : hGSH ratio
WT roots 3 weeks	96 ± 5	46 ± 2	2.1
WT nodules 3 weeks	244 ± 6	62 ± 3	4.0
sym40-1 roots 3 weeks	66 ± 9	43 ± 7	1.5
sym40-1 nodules 3 weeks	117 ± 12	40 ± 3	2.9
sym33-3 roots 3 weeks	75 ± 5	49 ± 10	1.5
sym33-3 nodules 3 weeks	258 ± 21	84 ± 1	3.0
sym33-2 roots 3 weeks	84 ± 10	58 ± 8	1.4
sym33-2 nodules 3 weeks	103 ± 5	49 ± 3	2.1
	83 ± 6	50 ± 5	
WT roots 1 weeks	131 ± 8	53 ± 7	1.6
WT nodules 1 weeks	56 ± 4	32 ± 3	2.4
WT roots 2 weeks	166 ± 13	31 ± 5	1.7
WT nodules 2 weeks	96 ± 5	46 ± 2	5.4

Data are means ± SEM of four or five replicates. High-performance liquid chromatography–high-resolution mass-spectrometry was used to define the profile of thiols and quantify their amount (nmol/g) in the samples.
GSH, glutathione; hGSH, homoglutathione.

Supplementary Table 3. The relative expression levels of genes in roots with nodules of treated wild-type and mutant plants (lists 1, 3); statistical analysis of gene expression in experiments with treatments (lists 2, 4)
Excel file

Supplementary Table 4. Nodule number and thiol content in 2-week-old roots with nodules from wild-type and mutant pea plants treated with 0.1 mM buthionine sulphoximine (BSO) and 0.5 mM glutathione (GSH)

	Number of nodules*	Reduction in the number of nodules (%)	GSH content (%)**	hGSH content (%)**
WT	34.0 ± 7.0		100	100
WT +BSO	18.5 ± 3.8	46	6	4
WT +BSO+GSH	37.0 ± 5.4		237	88
sym40-1	25.0 ± 1.0		100	100
sym40-1 +BSO	18.5 ± 3.5	26	6	1
sym40-1 +BSO+GSH	31.0 ± 2.6		103	113
sym33-3	13.7± 8.5		100	100
sym33-3 +BSO	0.3 ± 0.3	98	3	1
sym33-3 +BSO+GSH	4.5 ± 2.5	50	37	18
sym33-2	2.5 ± 1.5		100	100
sym33-2 +BSO	1.3 ± 0.7	50	4	2
sym33-2 +BSO+GSH	2.8 ± 0.9		290	49

*The value represents the mean ± SEM of three replicates.

** For each genotype, the GSH or hGSH content (in nmol/g) in untreated roots with nodules was set to 100%.
High-performance liquid chromatography–high-resolution mass-spectrometry was used to define the profile of thiols and quantify their relative amounts. Experiments were performed with three replicates of a minimum of 10 plants per treatment. hGSH, homoglutathione.

Supplementary Table 5. The number of abnormal symbiosomes in 2-week-old *sym40-1* nodules from pea treated with 0.1 mM buthionine sulphoximine (BSO) and 0.5 mM glutathione (GSH)

	Number of all cells in the area*	Number of infected cells in the area*	Number of cells with abnormal symbiosomes in the area*
sym40-1	24 ± 1,1	19 ± 1,3	0 ± 0,1
sym40-1 +BSO	22,3 ± 0,5	18,2 ± 0,5	8,5 ± 0,3****
sym40-1 +BSO +GSH	30,5 ± 2,4	19,5 ± 2,8	0

*The value represent means ± SEM of 25 (for control; +GSH) and 83 (for +BSO) field of view of two replicates.
****Statistically significant difference compare to control and *sym40-1* +BSO+GSH variants

Supplementary Table 6. Thiol content in 2-week-old roots with nodules from wild-type and mutant pea plants treated with 0.1 mM or 1 mM glutathione (GSH)

	GSH (%)	hGSH (%)
WT control	100	100
WT + 0.1 mM GSH	205	97
WT + 1 mM GSH	322	300
sym40-1 control	100	100
sym40-1 + 0.1 mM GSH	92	126
sym40-1 + 1 mM GSH	284	260
sym33-3 control	100	100
sym33-3 + 0.1 mM GSH	128	66
sym33-3 + 1 mM GSH	157	206

For each genotype, the GSH or hGSH content (in nmol/g) in untreated roots with nodules was set to 100%.
High-performance liquid chromatography–high-resolution mass-spectrometry was used to define the profile of thiols and quantify their relative amounts. Experiments were performed with three replicates of a minimum of 10 plants per treatment.
